# Additive interaction of family medical history of cardiovascular diseases with hypertension and diabetes on the diagnosis of cardiovascular diseases among older adults in India

**DOI:** 10.3389/fcvm.2024.1386378

**Published:** 2024-12-05

**Authors:** Waquar Ahmed, Priyanka Dixit, Shiva Halli

**Affiliations:** ^1^ School of Health Systems Studies, Tata Institute of Social Sciences, Mumbai, India; ^2^ Department of Community Health Sciences, Faculty of Health Sciences, University of Manitoba, Winnipeg, MB, Canada

**Keywords:** additive interaction, synergistic effect, family medical history, hypertension, diabetes, cardiovascular diseases, older adults, India

## Abstract

**Introduction:**

The present study aimed to examine the additive interaction of family medical history of cardiovascular disease (CVD) and self-reported hypertension and diabetes on the diagnosis of CVD among older adults aged 45 years and above in India. A family medical history of CVD in individuals with hypertension and diabetes could identify a subpopulation with a higher risk of CVD.

**Methods:**

The study used the data from the Longitudinal Ageing Study in India (LASI) Wave 1 (2017–2018). The total sample size for the study was 58,734 older adults aged 45 years and above. An additive model was applied to determine the additive interaction effect of the family medical history of CVD with hypertension and diabetes on the diagnosis of CVD by calculating three different measures of additive interaction: the relative excess risk due to interaction (RERI), attribution proportion due to interaction (AP), and synergy index (S).

**Results:**

The prevalence of CVD was higher among hypertensive individuals with a family medical history of CVD (18.6%) than individuals without the coexistence of family medical history of CVD and hypertension (4.7%), and hypertensive individuals without family medical history of CVD (11.3%). On the other hand, the prevalence of CVD was higher among individuals with diabetes and family history of CVD (20.5%) than individuals without the coexistence of family history of CVD and diabetes (5.0%). Individuals with parental and sibling medical history had two times higher odds of having chronic heart diseases and strokes, respectively than those without parental and sibling history. In the adjusted model, RERI, AP, and S for CVD were 2.30 (95% CI: 0.87–3.74), 35% (0.35; 95% CI: 0.20–0.51), and 1.71 (95% CI: 1.27–2.28) respectively, demonstrating significant positive interaction between family medical history and hypertension on the diagnosis of cardiovascular diseases.

**Conclusions:**

The present study revealed that in the additive model, the interaction effects of family medical history and hypertension were significantly positive on cardiovascular diseases even after adjustment with potential confounding factors. Therefore, it is crucial to consider the presence of family medical history of CVD among individuals with hypertension and diabetes measured in research and clinical practice.

## Introduction

Family history of non-communicable diseases is a significant non-modifiable risk factor for various conditions, including coronary heart disease (1), stroke (2), diabetes (3), hypertension (4), as well as breast cancer, lung cancer, colorectal cancer, prostate cancer, and ovarian cancer (5). Family history is a significant indicator of genetic factors, and it is frequently employed as an alternative measure to investigate the association between genetic factors and diseases (6–9). According to estimates by the American Heart Association, approximately 13.8% of American adults aged 20 years and older reported having a family history of heart disease (10). Existing literature suggests that ischemic stroke and coronary heart disease are heritable (10–12). Additionally, one prior study has shown that a family medical history of premature coronary heart disease (angina, myocardial infarction, angioplasty, or bypass surgery) was significantly associated with a nearly 50% increase in the lifetime risk for both coronary heart disease and cardiovascular disease mortality (13). A recent study demonstrated that heart disease was more prevalent among individuals with hypertension, diabetes, and family medical history of heart diseases (14).

High blood pressure, beyond its well-established association with coronary heart disease and stroke, is a significant risk factor for heart failure, atrial fibrillation, chronic kidney disease, heart valve diseases, aortic syndromes, and dementia (10, 15–17). Additionally, in a meta-analysis, it has been shown that 20 mm Hg increase in systolic blood pressure and 10 mm Hg increase in diastolic blood pressure was significantly associated with a twofold risk of mortality due to stroke, ischemic heart disease (IHD) and other vascular diseases (18, 19). In a previous study, it was reported that relying solely on hypertension as a predictor of individual risk of CVD is insufficient. Hereditary factors may elucidate why some specific hypertensive individuals are more susceptible to CVD and why certain ethnic groups have a higher incidence of hypertension-related CVD (20).

In the ageing population, diabetes is a significant risk factor for the development of cardiovascular disease (CVD) (21). Multiple studies demonstrated that individuals aged 40 to 75 with diabetes face an intermediate or high risk of atherosclerotic cardiovascular events (18, 21–23). Furthermore, the risk of CVD progressively increases with higher fasting plasma glucose levels, even before reaching the diagnostic threshold for diabetes (24).

The presence of family history of CVD among individuals with hypertension and diabetes is relatively common, and their coexistence may act synergistically on the risk of CVD. There is a substantial gap in the literature concerning the interaction effect of family history with hypertension and diabetes on cardiovascular diseases in low and middle-income countries (LMICs), especially in India (20, 25). Thus, the current study aimed to examine the additive interaction of family medical history of CVD with self-reported hypertension and diabetes on cardiovascular diseases. The findings on the significance of the interaction effect are essential for developing an effective holistic disease management strategy rather than disease-specific programs.

## Materials and methods

### Data

This study used the data from the Longitudinal Ageing Study in India (LASI) Wave 1 (2017–2018), a large-scale sample survey for the current analysis. The survey has been conducted under the Ministry of Health and Family Welfare (MoHFW), Government of India. International Institute for Population Sciences (IIPS) collaborated with Harvard T. H. Chan School of Public Health and the University of Southern California. The survey collected information on the health, economic, social, and demographic aspects of India's ageing population as well as its consequences. The LASI is a nationally representative survey that included 72,250 individuals who were 45 years of age or older along with their spouses (irrespective of age) in all Indian states and union territories of India except Sikkim. The LASI employs a multistage stratified area probability cluster sampling to select the eventual units of observation. The LASI provides information on chronic health conditions, biomarkers, symptom-based health conditions, and functional and mental health. The LASI survey was carried out using a three-stage and four-stage sampling design in rural and urban areas, respectively. In each state/UT, Primary Sampling Units (PSUs) were chosen in the first stage, while villages in rural areas and wards in urban areas were chosen in the selected PSUs in the second stage. Households were selected from each identified village in the third stage; however, sampling in urban areas required an additional stage, which comprised randomly selecting one Census Enumeration Block (CEB) in each urban area. From each CEB, households were selected in the fourth stage. The main goal was to select a representative sample at each stage of sample selection.

**Figure 1:**
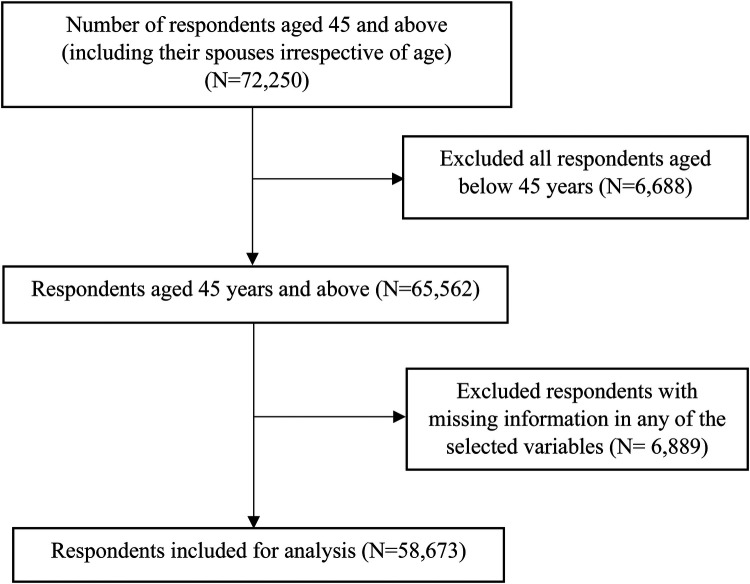
Study sample flowchart.

The LASI used computer-assisted personal interview (CAPI) technology for the data collection. This method required field teams to be outfitted with laptop computers pre-loaded with survey questions asked of respondents in a face-to-face interview. The report contains considerable information on the survey design, data collection, and methodology. The present study is based on eligible respondents aged 45 years and above. The present study is based on 65,562 respondents aged 45 years and above. After excluding 6,828 cases with missing information in any of the selected variables (including body mass index: 6,489, hypertension, diabetes, and CVD: 185 and selected covariates), the total sample size for the analysis was 58,734 respondents (Figure 1).

## Measures

### Outcome variables

The outcome variables were self-reported chronic heart diseases and stroke. In the study, respondents were asked, “Has any health professional ever diagnosed you with chronic heart diseases such as coronary heart disease (heart attack or Myocardial Infarction), congestive heart failure, or other chronic heart problems?”. Respondents were also asked, “Has any health professional ever diagnosed you with a stroke?”. The responses were coded as no and yes for each chronic disease and therefore considered as self-reported. For the analysis, chronic heart diseases and stroke were combined to create CVD as one variable and coded it as no and yes.

### Key explanatory variable

The main explanatory variables were self-reported hypertension, diabetes and family medical history. In the study, respondents were asked, “Has any health professional ever diagnosed you with high blood pressure or hypertension?”. Respondents were also asked, “Has any health professional ever diagnosed you with diabetes?”. The responses were coded as no and yes for each chronic disease. In the LASI, to understand the genetic risk factors for cardiovascular diseases, information was collected about the respondent's family medical history; family medical history of the father, mother, brother, and sister were selected for the analysis.

### Other covariates

Age was coded as 45–54 years, 55–64 years, 65–74 years and 75 + years. Sex was coded as male and female. Education was recoded as no education, primary, secondary, and higher. Marital status was coded as currently married, widowed, and others, not a union. Working status was coded as never worked, currently working, and currently not working. Alcohol use was coded as “no” and “yes”; smoking and chewing tobacco were coded as “never”, “former, and “current” The BMI was computed by dividing the weight (in kilograms) by the square of the height (in meters). BMI was coded according to the criteria of the World Health Organisation's classification; as underweight (<18.5 kg/m^2^), normal weight (18.5–24.9 kg/m^2^), overweight (25.0–29.9 kg/m^2^), and obesity (≥30.0 kg/m^2^), for the analysis, overweight and obesity were combined (26). The monthly per capita expenditure quintile (MPCE) or consumption quintile was categorized into five quintiles, poorest, poor, middle, rich, and richest. Religion was categorized as Hindu, Muslim, Christian, and Others. The social group (caste) was categorized as Scheduled Castes (SC), Scheduled Tribes (ST), Other Backward Classes (OBC), and others. The “other” category in caste is identified as non SC, ST, and non OBC caste. The place of residence was coded as urban and rural. The regions were categorized as North, Central, East, Northeast, West, and South. difficulty in activities of daily living (ADL) and difficulty in instrumental activities of daily living (IADL) was coded as “no” and “yes” (27).

To investigate the level of physical activities, respondents were asked the type and amount of physical activity involved in daily life. For vigorous activities, respondents were asked “*How do you often take part in sports or vigorous activities such as running or jogging, swimming, going to health centre/gym, cycling, digging with a spade or shovel, heavy lifting, chopping, farm work, fast bicycling, and cycling with loads?”.* For moderate activities, respondents were asked “*How do you often take part in sports or activities that are moderately energetic such as cleaning house, washing clothes by hand, fetching water or wood, drawing water from a well, gardening, bicycling at a regular pace, walking at a moderate pace, dancing, floor or stretching exercises?”.* Every day, more than once a week, once a week, one to three times per month, and hardly ever or never were the possible responses for moderate and vigorous physical activities. For both moderate and vigorous activities, respondents were also asked “on the days you did activity, how much time did you usually spend doing any activity?”.

Weekly time spent for both moderate and vigorous physical activity was calculated (Moderate physical activity: Those who performed at least 150 min of moderate-intensity physical activity throughout the week. Vigorous physical activity: Those who performed at least 75 min of vigorous-intensity physical activity throughout the week). For both moderate and vigorous activities, respondent was categorized as physically active (*engagement more than once a week*) or physically inactive (*engagement once a week or less often*) based on the responses. Then physical activity variable was created as Physically active (Those who were either engaged in moderate physical activity or vigorous physical activity) and otherwise physically inactive (28–31).

### Statistical analysis

Unadjusted and adjusted estimates were calculated using multivariable logistic regression models to assess the joint effect of family medical history of CVD with hypertension and diabetes on the diagnosis of CVD. An additive model was applied to determine the additive interaction effect by calculating three different measures of additive interaction: the relative excess risk due to interaction (RERI), attribution proportion due to interaction (AP), and synergy index (S) (30, 32, 33). Survey weights were applied to calculate the three different measures of additive interaction in the unadjusted and adjusted additive model. Confidence intervals of these three measures were estimated, and *P* < 0.05 was considered statistically significant.

The interaction measures on an additive scale are defined as:

RERI (Relative excess risk due to interaction) = OR11 - OR10 - OR01 + 1

AP (Attributable proportion to interaction) = RERI/OR11

S (Synergy index) = (OR11 – 1)/(OR10 – 1) + (OR01 – 1)

RERI =0 indicates no interaction, RERI > 0 indicates positive interaction, RERI < 0 indicates negative interaction, AP = 0 indicates no interaction, AP > 0 indicates positive interaction, AP < 0 indicates negative interaction, S = 1 indicates no interaction, S > 1 indicates positive interaction, S < 1 indicates negative interaction (30, 32–34).

Interaction on an additive scale means that the combined effect of two exposures is larger (or smaller) than the sum of the individual effects of the two exposures. The RERI is a measure of departure from additivity, which reflects the excess risk of the outcome (total combined effect) due to the interaction between two factors. The AP, on the other hand, is a measure of the proportion of the combined effect that is attributable to the interaction between two factors. Finally, the S is a measure of the ratio between combined effect and individual effects, with a value greater than 1 indicating a synergistic interaction (32–34).

Bivariate analysis (cross-tabulation) was conducted to examine the prevalence of chronic heart diseases, stroke and CVD with selected variables. A chi-square test and bivariate analysis (cross-tabulation) were also conducted to examine the prevalence of chronic heart diseases, stroke and CVD with respect to the joint effect of family medical history of chronic heart diseases, stroke and CVD with hypertension and diabetes. Additionally, multivariable binary logistic regression analysis (35) was used to establish the association between cardiovascular diseases and main explanatory variables (hypertension, diabetes and family medical history). The binary logistic regression model is usually put into a more compact form as follows:

$$\mathrm{Logit}\;[P(Y=1)]=\beta_{0}+\beta \;*\;X$$

The parameter $\beta_{0}$ estimates the log odds of outcome variables for the reference group, while β estimates the maximum likelihood, the differential log odds of outcome variables associated with a set of predictors X, compared to the reference group. In this study, the multivariable logistic regression and additive interaction models were adjusted for potential confounding factors, including age, sex, education, working status, marital status, residence, MPCE, religion, caste, region, physical inactivity, smoking, chewing tobacco, alcohol consumption, ADL, IADL, and body mass index (BMI). The survey weights were applied during the analysis to account for sample clustering and present population estimates. All the analyses were conducted using Stata version 14.1 (36).

## Results

Table 1 presents the sample characteristics. A proportion of 34.5% of the participants were 65 years and older in this study. About 54% of the sample population was female. A large proportion of the sample (50.5%) had no education, and 73.9% were in marital union during the survey. Further majority of the sample population belonged to rural areas (70.1%).

**Table 1:** Sample characteristics, longitudinal ageing study in India.

Variables	*N*	%
Sociodemographic variables		
Age group		
45–54	21,790	35.09
55–64	18,232	30.46
65–74	13,025	24.03
75+	5,626	10.42
Sex		
Male	27,177	45.90
Female	31,496	54.10
Education level		
No education	27,521	50.45
Primary	14,661	23.43
Secondary	10,865	16.29
Higher	5,626	9.83
Working status		
Never worked	16,040	25.98
Currently working	27,357	47.28
Currently not working	15,276	26.74
Marital status		
Currently married	43,977	73.94
Widowed	12,824	23.24
D/S/D/Others	1,872	2.82
Place of residence		
Rural	38,395	70.11
Urban	20,278	29.89
Caste		
Scheduled caste	9,878	19.41
Scheduled tribe	10,266	8.64
OBC	22,157	45.47
Others	16,372	26.47
MPCE quintiles		
Poorest	11,549	20.98
Poorer	11,859	21.28
Middle	11,867	20.47
Richer	11,844	19.69
Richest	11,554	17.57
Region		
North	10,816	12.71
Central	8,062	21.08
East	10,617	23.91
Northeast	7,583	3.38
West	7,705	15.94
South	13,890	22.98
Religion		
Hindu	43,060	82.49
Muslim	6,951	11.05
Christian	5,878	2.95
Others	2,784	3.51
Lifestyle factors		
Smoking tobacco		
Never	47,880	82.74
Former	2,671	3.78
Current	8,122	13.48
Chewing tobacco		
Never	45,799	76.41
Former	1,436	2.38
Current	11,438	21.20
Alcohol Use		
No	48,105	84.83
Yes	10,568	15.17
BMI categories		
Normal	30,665	51.57
Underweight	10,859	21.34
Overweight/obese	17,149	27.09
Physical inactivity		
Active	37,904	65.77
Inactive	20,769	34.23
Morbidity		
Difficulty in ADL		
No	50,737	84.47
Yes	7,936	15.53
Difficulty in IADL		
No	39,646	63.47
Yes	19,027	36.53
Hypertension		
No	41,918	73.00
Yes	16,755	27.00
Diabetes		
No	51,188	88.08
Yes	7,485	11.92
Family medical history		
Family history		
No	52,313	88.39
Yes	6,360	11.61
Parental FH		
No	54,134	91.33
Yes	4,539	8.67
FH of father		
No	55,943	94.46
Yes	2,730	5.54
FH of mother		
No	56,465	96.12
Yes	2,208	3.88
Sibling FH		
No	56,403	96.27
Yes	2,270	3.73
FH of brother		
No	56,936	97.10
Yes	1,737	2.90
FH of sister		
No	58,017	98.96
Yes	656	1.04
Total	58,673	100.00

%: weighted percentages to account for survey design and to provide national population estimates; ADL, activities of daily living; IADL, instrumental activities of daily living; MPCE, monthly per capita consumption expenditure; FH, family medical history (family medical history of chronic heart disease, stroke and CVD).

Table 2 depicts the prevalence of chronic heart diseases, stroke and cardiovascular diseases (CVD) among adults aged 45 years and above. The overall prevalence of chronic heart diseases, stroke and CVD was 3.8% (95% CI: 3.4, 4.4), 1.7% (95% CI: 1.5, 1.8), and 5.3% (95% CI: 4.8, 5.8), respectively. The prevalence of chronic heart diseases (6.0%) was higher among those 65–74 years of age group, while the prevalence of stroke (2.8%) was higher among individuals 75 years of age and above. The prevalence of chronic heart diseases (4.2% vs. 3.6%) and stroke (2.1% vs. 1.3) were higher among males than female respondents. The prevalence of chronic heart diseases (5.8% vs. 3.0%), stroke (1.9% vs. 1.6%), and CVD (7.3% vs. 4.4%) was higher among those living in urban areas than individuals living in rural areas. Moreover, the results show that the prevalence of chronic heart diseases (4.8% vs. 3.4%), stroke (2.6% vs. 1.2%) and CVD (7.0% vs. 4.4%) were higher among physically inactive participants than those who were physically active. Chronic heart diseases (8.0% vs. 3.5%), stroke (4.9% vs. 1.5%), and CVD (9.2% vs. 4.8%) were more prevalent among those who had a family history of respective chronic diseases than individuals without family medical history. The prevalence of chronic heart diseases and stroke was 9.3% and 3.8% among individuals with hypertension. Further, the prevalence of chronic heart diseases and stroke was 10.7% and 3.8% among individuals with diabetes. The prevalence of cardiovascular diseases was higher among individuals with hypertension (12.4% vs. 2.7%) and diabetes (13.6% vs. 4.1%) than those without hypertension and diabetes.

**Table 2:** Prevalence of chronic heart diseases, stroke, and cardiovascular diseases with respect to various background characteristics among older adults in India.

Background characteristics	Chronic heart diseases		Stroke		CVD	
	*N*	% (95% CI)	*N*	% (95% CI)	*N*	% (95% CI)
Sociodemographic variables						
Age group						
45–54	420	2.1 (1.6,2.6)	176	0.9 (0.7,1.1)	576	2.8 (2.4,3.3)
55–64	686	3.6 (3.3,4.1)	268	1.6 (1.3,1.9)	917	4.9 (4.5,5.4)
65–74	703	6 (4.4,8.1)	321	2.5 (2.1,2.9)	984	8.1 (6.5,10.2)
75+	325	5.5 (4.2,7.2)	160	2.8 (2.3,3.5)	459	8.0 (6.6,9.7)
Sex						
Male	1,151	4.2 (3.7,4.6)	555	2.1 (1.9,2.4)	1,630	6 (5.5,6.5)
Female	983	3.6 (2.8,4.6)	370	1.3 (1.1,1.5)	1,306	4.7 (3.9,5.7)
Education level						
No education	719	2.8 (2.4,3.2)	373	1.4 (1.2,1.7)	1,055	4.1 (3.7,4.5)
Primary	600	4.3 (3.6,5.1)	258	2 (1.7,2.4)	829	6 (5.3,6.8)
Secondary	522	6.0 (3.8,9.3)	190	2 (1.7,2.5)	678	7.6 (5.4,10.7)
Higher	293	4.6 (3.7,5.6)	104	1.5 (1.1,2.0)	374	5.7 (4.8,6.9)
Working status						
Never worked	652	4.8 (3.3,6.9)	209	1.4 (1.1,1.6)	831	5.9 (4.4,8.0)
Currently working	583	2.2 (1.9,2.5)	227	0.8 (0.7,1.0)	783	2.9 (2.6,3.2)
Currently not working	899	5.8 (5.1,6.6)	489	3.4 (3.0,3.9)	1,322	8.8 (8.0,9.6)
Marital status						
Currently married	1,596	3.7 (3.3,4.0)	677	1.6 (1.4,1.8)	2,173	5 (4.6,5.4)
Widowed	492	4.7 (3.1,7.1)	231	2.1 (1.7,2.5)	701	6.6 (4.9,8.8)
D/S/D/Others	46	1.5 (0.9,2.4)	17	0.6 (0.3,1.3)	62	2.1 (1.4,3.1)
Place of residence						
Rural	1,060	3.0 (2.7,3.3)	535	1.6 (1.4,1.8)	1,543	4.4 (4.1,4.8)
Urban	1,074	5.8 (4.4,7.6)	390	1.9 (1.6,2.2)	1,393	7.3 (5.9,9.0)
Caste						
Scheduled caste	302	3.4 (2.6,4.3)	169	2 (1.6,2.4)	455	5.1 (4.2,6.0)
Scheduled tribe	154	1.2 (0.9,1.7)	117	1 (0.7,1.4)	258	2 (1.6,2.5)
OBC	816	3.9 (3.0,5.1)	323	1.4 (1.2,1.6)	1,107	5.2 (4.2,6.3)
Others	862	4.9 (4.5,5.4)	316	2.1 (1.8,2.5)	1,116	6.7 (6.1,7.3)
MPCE quintiles						
Poorest	297	2.7 (2.2,3.3)	123	1.2 (0.9,1.5)	405	3.7 (3.2,4.3)
Poorer	321	2.9 (2.3,3.6)	177	1.6 (1.3,1.9)	488	4.4 (3.7,5.1)
Middle	410	3.4 (2.9,4.1)	194	1.7 (1.4,2.0)	574	4.8 (4.2,5.6)
Richer	477	3.8 (3.3,4.3)	196	1.7 (1.4,2.1)	645	5.2 (4.6,5.8)
Richest	629	6.9 (4.8,10.0)	235	2.3 (1.9,2.7)	824	8.8 (6.6,11.7)
Region						
North	525	4.1 (3.6,4.6)	148	1.5 (1.2,1.9)	645	5.3 (4.7,5.9)
Central	137	1.8 (1.5,2.3)	98	1.2 (0.9,1.5)	225	2.8 (2.4,3.4)
East	380	4.1 (3.5,5.0)	200	2.1 (1.8,2.5)	545	5.9 (5.1,6.7)
Northeast	140	2.7 (2.1,3.4)	97	1.3 (0.9,1.8)	228	3.9 (3.2,4.7)
West	307	4.9 (4.2,5.7)	165	2.4 (2.0,2.9)	451	7 (6.2,7.9)
South	645	4.7 (3.0,7.1)	217	1.3 (1.1,1.6)	842	5.9 (4.2,8.2)
Religion						
Hindu	1,473	3.6 (3.1,4.3)	652	1.6 (1.4,1.8)	2,036	5 (4.4,5.7)
Muslim	427	5.3 (4.6,6.1)	132	2 (1.6,2.5)	536	6.9 (6.1,7.8)
Christian	142	4.2 (2.9,6.1)	78	1.4 (0.8,2.4)	213	5.6 (4.0,7.7)
Others	92	3.6 (2.6,5.0)	63	2.7 (1.9,4.0)	151	6.3 (4.9,8.0)
Lifestyle factors						
Smoking tobacco						
Never	1,650	3.7 (3.1,4.3)	694	1.5 (1.4,1.7)	2,257	5 (4.4,5.6)
Former	235	9.8 (7.3,13.1)	88	3.7 (2.6,5.2)	305	12.7 (10.0,15.9)
Current	249	3.2 (2.6,3.9)	143	2 (1.6,2.5)	374	5 (4.3,5.8)
Chewing tobacco						
Never	1,752	4.0 (3.4,4.7)	733	1.7 (1.5,1.9)	2,379	5.5 (4.9,6.2)
Former	90	8.0 (4.9,13.0)	46	3.4 (2.4,5.0)	131	11 (7.5,15.7)
Current	292	2.7 (2.3,3.2)	146	1.4 (1.1,1.8)	426	3.9 (3.4,4.4)
Alcohol use						
No	1,784	3.9 (3.4,4.6)	712	1.6 (1.4,1.8)	2,397	5.3 (4.7,6.0)
Yes	350	3.3 (2.8,3.9)	213	2.1 (1.7,2.5)	539	5.1 (4.5,5.8)
BMI categories						
Normal	1,047	3.6 (3.2,4.0)	494	1.8 (1.6,2.0)	1,481	5.2 (4.7,5.6)
Underweight	213	2.1 (1.7,2.5)	123	1.3 (1.0,1.6)	326	3.2 (2.8,3.7)
Overweight/obese	874	5.7 (4.2,7.7)	308	1.7 (1.5,2.1)	1,129	7.1 (5.6,9.0)
Physical inactivity						
Active	1,167	3.4 (2.7,4.2)	421	1.2 (1.0,1.3)	1,540	4.4 (3.7,5.2)
Inactive	967	4.8 (4.2,5.4)	504	2.6 (2.3,3.0)	1,396	7 (6.4,7.7)
Morbidity						
Difficulty in ADL						
No	1,642	3.4 (2.8,4.0)	579	1.2 (1.1,1.4)	2,151	4.4 (3.9,5.1)
Yes	492	6.3 (5.4,7.5)	346	4.1 (3.5,4.7)	785	9.8 (8.7,11.0)
Difficulty in IADL						
No	1,224	3.0 (2.7,3.4)	389	1 (0.9,1.1)	1,563	3.9 (3.6,4.3)
Yes	910	5.2 (4.1,6.7)	536	2.8 (2.5,3.2)	1,373	7.7 (6.5,9.1)
Hypertension						
No	775	1.8 (1.6,2.1)	326	0.9 (0.8,1.0)	1,080	2.6 (2.4,2.9)
Yes	1,359	9.3 (7.7,11.2)	599	3.8 (3.4,4.3)	1,856	12.4 (10.7,14.2)
Diabetes						
No	1,466	2.9 (2.7,3.2)	661	1.4 (1.2,1.5)	2,058	4.1 (3.9,4.4)
Yes	668	10.7 (7.5,14.9)	264	3.8 (3.2,4.6)	878	13.6 (10.4,17.6)
Family medical history						
Family history						
No	1,724	3.5 (3.0,4.1)	789	1.5 (1.3,1.6)	2,262	4.8 (4.2,5.4)
Yes	410	8.0 (6.7,9.5)	136	4.9 (3.9,6.2)	674	9.2 (8.1,10.4)
Parental FH						
No	1,868	3.6 (3.1,4.2)	830	1.5 (1.4,1.7)	2,475	4.9 (4.4,5.5)
Yes	266	7.9 (6.2,9.9)	95	4.6 (3.5,6.0)	461	8.9 (7.6,10.5)
FH of father						
No	1,979	3.7 (3.2,4.3)	870	1.6 (1.4,1.7)	2,648	5.1 (4.5,5.6)
Yes	155	8.2 (5.9,11.1)	55	4.7 (3.3,6.7)	288	9 (7.2,11.1)
FH of mother						
No	1,998	3.8 (3.3,4.3)	877	1.6 (1.5,1.8)	2,658	5.1 (4.6,5.7)
Yes	136	7.3 (5.6,9.4)	48	4.5 (3.0,6.5)	278	9.1 (7.6,10.9)
Sibling FH						
No	1,950	3.7 (3.2,4.3)	877	1.6 (1.5,1.8)	2,691	5.1 (4.5,5.6)
Yes	184	9.3 (7.5,11.6)	48	6.4 (4.3,9.4)	245	10.9 (9.2,12.9)
FH of brother						
No	1,991	3.7 (3.2,4.3)	887	1.6 (1.5,1.8)	2,711	5.1 (4.6,5.7)
Yes	143	10.2 (8.0,13.0)	38	6.9 (4.4,10.6)	225	11.6 (9.6,14.1)
FH of sister						
No	2,082	3.8 (3.3,4.4)	911	1.7 (1.5,1.8)	2,849	5.2 (4.7,5.8)
Yes	52	7.9 (4.9,12.5)	14	5.4 (2.6,10.8)	87	9.2 (6.6,12.9)
Total	2,134	3.8 (3.4,4.4)	925	1.7 (1.5,1.8)	2,936	5.3 (4.8,5.8)

%: weighted percentages to account for survey design and to provide national population estimates; CVD, cardiovascular diseases; ADL, Activities of daily living; IADL, Instrumental activities of daily living, MPCE, Monthly per capita consumption expenditure; FH, family medical history (family medical history of chronic heart disease, stroke and CVD).

Table 3 illustrates the prevalence of chronic heart diseases, stroke and CVD with respect to the key predictor variables among individuals aged 45 and above.

**Table 3:** Prevalence of chronic heart diseases, stroke, and cardiovascular diseases with respect to combined effect of the key predictor variables among older adults in India.

Variables	Chronic heart diseases			Stroke			Cardiovascular diseases		
	*N*	% (95% CI)	*P*-value	*N*	% (95% CI)	*P*-value	*N*	% (95% CI)	*P*-value
FH and hypertension			0.000			0.000			0.000
No	1,858	3.5 (3.0,4.1)		831	1.5 (1.4,1.7)		2,486	4.7 (4.2,5.3)	
Yes	276	16.2 (13.4,19.5)		94	9.4 (7.2,12.3)		450	18.6 (16.1,21.3)	
FH and diabetes			0.000			0.000			0.000
No	1,994	3.7 (3.2,4.3)		882	1.6 (1.5,1.8)		2,709	5.0 (4.5,5.6)	
Yes	140	18.2 (14.0,23.3)		43	10.7 (7.0,16.1)		227	20.5 (16.8,24.8)	
FH#Hypertension			0.000			0.000			0.000
0.FH#0.hypertension	641	1.7 (1.5,2.0)		284	0.8 (0.7,0.9)		856	2.4 (2.2,2.7)	
0.FH#1.hypertension	1,083	8.6 (6.9,10.7)		505	3.3 (2.9,3.8)		1,406	11.3 (9.5,13.5)	
1.FH#0.hypertension	134	3.6 (2.7,4.8)		42	2.5 (1.7,3.7)		224	4.4 (3.6,5.4)	
1.FH#1.hypertension	276	16.2 (13.4,19.5)		94	9.4 (7.2,12.3)		450	18.6 (16.1,21.3)	
FH#Diabetes			0.000			0.000			0.000
0.FH#0.diabetes	1,196	2.6 (2.4,2.9)		568	1.2 (1.1,1.4)		1,611	3.7 (3.4,4.0)	
0.FH#1.diabetes	528	10.0 (6.7,14.8)		221	3.4 (2.8,4.1)		651	12.7 (9.1,17.4)	
1.FH#0.diabetes	270	6.4 (5.2,7.9)		93	4.1 (3.1,5.3)		447	7.5 (6.5,8.8)	
1.FH#1.diabetes	140	18.2 (14.0,23.3)		43	10.7 (7.0,16.1)		227	20.5 (16.8,24.8)	
Total	2,134	3.8 (3.4,4.4)		925	1.7 (1.5,1.8)		2,936	5.3 (4.8,5.8)	

FH, family medical history.

### The prevalence of CVD, chronic heart diseases and stroke based on the presence or absence of family medical history and hypertension

The results indicate that the prevalence of CVD was higher among hypertensive individuals with family medical history of CVD (18.6%, 95% CI: 16.1–21.3) than individuals without the coexistence of family medical history of CVD and hypertension (4.7%, 95% CI: 4.2–5.3), hypertensive individuals without a family medical history of CVD (11.3%, 95% CI: 9.5–13.5), individuals with the presence of family medical history of CVD and without hypertension (4.4%, 95% CI: 3.6–5.4), and individuals without the presence of both family medical history of CVD and hypertension (2.4%, 95% CI: 2.2–2.7).

Moreover, the results indicate that the prevalence of chronic heart diseases and stroke was higher among hypertensive individuals with family medical history (16.2% and 9.4%) than those without coexistence of family medical history and hypertension (3.5% and 1.5%), hypertensive individuals without family medical history (8.6% and 3.3%), and individuals with family medical history and without hypertension (3.6% and 2.5%).

### The prevalence of CVD, chronic heart diseases and stroke based on the presence or absence of family medical history and diabetes

Our findings show that the prevalence of CVD was higher among individuals with the coexistence of diabetes and family history of CVD (20.5%, 95% CI: 16.8–24.8) than individuals without coexistence of a family history of CVD and diabetes (5.0%, 95% CI: 4.5–5.6), individuals with diabetes and without a family history of CVD (12.7%, 95% CI: 9.1–17.4), individuals with a family history of CVD and without diabetes (7.5%, 95% CI: 6.5–8.8), and individuals without the presence of both family medical history of CVD and diabetes (3.7%, 95% CI: 3.4–4.0).

Moreover, the results indicate that the prevalence of chronic heart diseases and stroke was higher among individuals with the coexistence of family medical history and diabetes (18.2% and 10.7%) than individuals without coexistence of family medical history and diabetes (3.7% and 1.6%), individuals with diabetes and without family medical history (10.0% and 3.4%), and individuals with a family medical history and without diabetes (6.4% and 4.1%).

**Figure 2:**
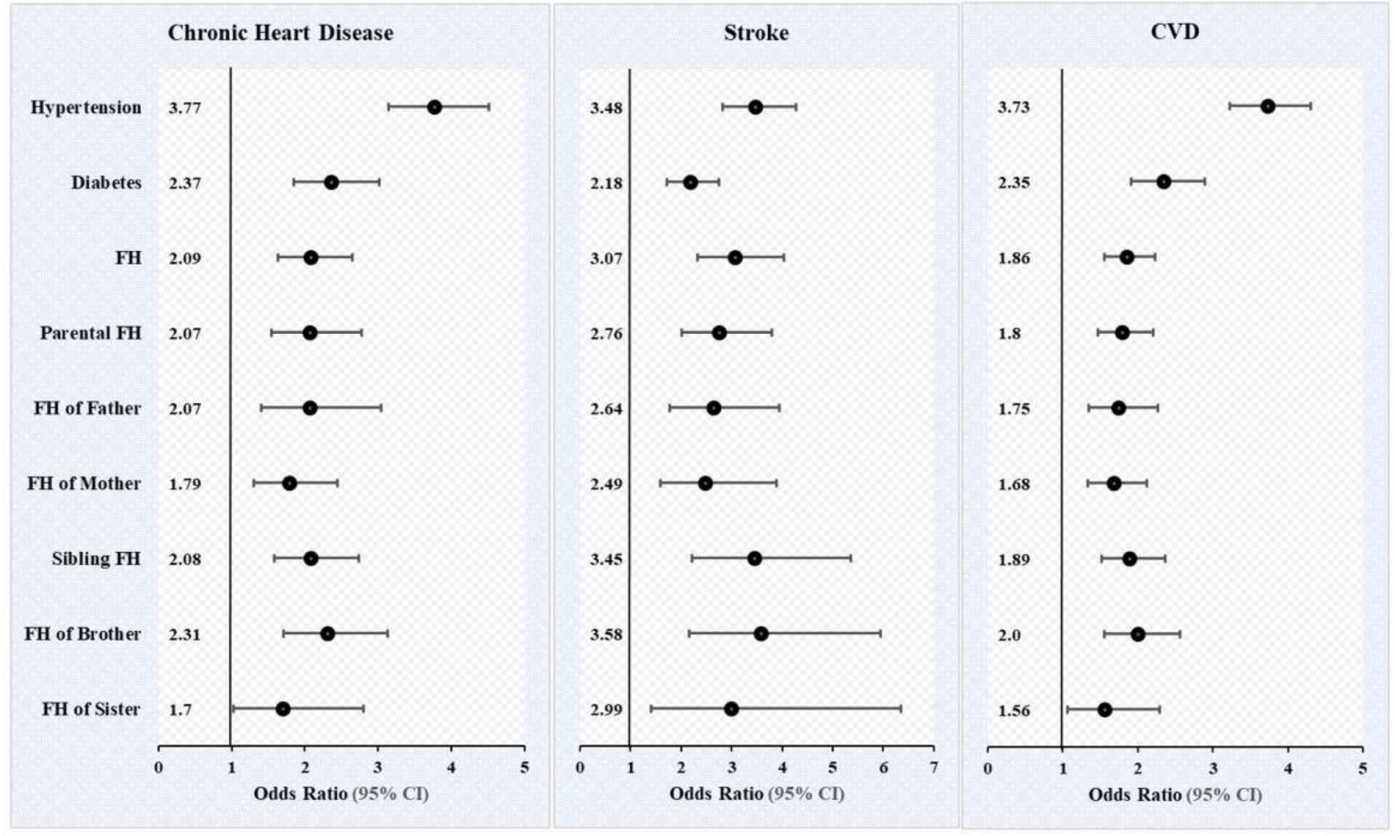
Multivariable logistic estimates for cardiovascular diseases according to key predictor variables among individuals aged 45 and above. Odds ratio, adjusted for age, sex, education, working, marital status, residence, MPCE, religion, caste, region, physical inactivity, smoking, chewing tobacco, alcohol consumption, body mass index, ADL and IADL. FH, family medical history (family history of chronic heart diseases, stroke, and cardiovascular diseases).

Table 4 presents the unadjusted and adjusted logistic regression estimates for chronic heart disease, stroke and cardiovascular diseases by hypertension, diabetes, and family medical history of cardiovascular diseases (any family history, parental history, father, mother, sibling history, brother and sister) among older adults aged 45 years and above in India. Figure 2 presents the adjusted logistic regression estimates for chronic heart disease, stroke and cardiovascular diseases by hypertension, diabetes, and family medical history of cardiovascular diseases (any family history, parental history, father, mother, sibling history, brother and sister). The result indicates that individuals with hypertension had 3.77 [Adjusted odds ratio (AOR): 3.77, CI: 3.14–4.51] and 3.48 (AOR: 3.48, CI: 2.82–4.28) times higher odds of chronic heart disease and stroke, respectively, than those without hypertension. Participants with diabetes had significantly higher odds of having chronic heart diseases and stroke than those without diabetes.

**Table 4:** Multivariable logistic estimates for cardiovascular diseases according to key predictor variables among individuals aged 45 years and above.

Key Variables	Chronic heart diseases		Stroke		Cardiovascular diseases	
	UOR 95% CI	AOR 95% CI	UOR 95% CI	AOR 95% CI	UOR 95% CI	AOR 95% CI
Hypertension						
No	Ref.	Ref.	Ref.	Ref.	Ref.	Ref.
Yes	5.51*** (4.32 7.02)	3.77*** (3.14 4.51)	4.41*** (3.63 5.34)	3.48*** (2.82 4.28)	5.18*** (4.29 6.26)	3.73*** (3.23 4.31)
Diabetes						
No	Ref.	Ref.	Ref.	Ref.	Ref.	Ref.
Yes	3.98*** (2.68 5.90)	2.37*** (1.85 3.02)	2.83*** (2.27 3.52)	2.18*** (1.72 2.75)	3.65*** (2.67 5.00)	2.35*** (1.91 2.89)
Family history of CVD						
	Ref.	Ref.	Ref.	Ref.	Ref.	Ref.
Yes	2.39*** (1.86 3.08)	2.09*** (1.64 2.65)	3.45*** (2.66 4.46)	3.07*** (2.33 4.03)	2.03*** (1.68 2.45)	1.86*** (1.56 2.23)
Parental FH						
No	Ref.	Ref.	Ref.	Ref.	Ref.	Ref.
Yes	2.27*** (1.70 3.05)	2.07*** (1.55 2.78)	3.10*** (2.30 4.18)	2.76*** (2.01 3.80)	1.90*** (1.54 2.34)	1.80*** (1.47 2.20)
FH of father						
No	Ref.	Ref.	Ref.	Ref.	Ref.	Ref.
Yes	2.32*** (1.60 3.36)	2.07*** (1.40 3.04)	3.04*** (2.06 4.50)	2.64*** (1.77 3.94)	1.86*** (1.43 2.42)	1.75*** (1.35 2.27)
FH of mother						
No	Ref.	Ref.	Ref.	Ref.	Ref.	Ref.
Yes	2.01*** (1.46 2.76)	1.79*** (1.31 2.44)	2.85*** (1.89 4.29)	2.49*** (1.60 3.89)	1.86*** (1.48 2.34)	1.68*** (1.33 2.12)
Sibling FH						
No	Ref.	Ref.	Ref.	Ref.	Ref.	Ref.
Yes	2.68*** (2.01 3.56)	2.08*** (1.58 2.74)	4.22*** (2.75 6.45)	3.45*** (2.22 5.36)	2.30*** (1.84 2.88)	1.89*** (1.52 2.37)
FH of Brother						
No	Ref.	Ref.	Ref.	Ref.	Ref.	Ref.
Yes	2.95*** (2.16 4.02)	2.31*** (1.71 3.13)	4.52*** (2.79 7.32)	3.58*** (2.16 5.94)	2.46*** (1.93 3.14)	2.00*** (1.56 2.56)
FH of Sister						
No	Ref.	Ref.	Ref.	Ref.	Ref.	Ref.
Yes	2.18** (1.28 3.69)	1.70* (1.03 2.80)	3.39** (1.59 7.27)	2.99** (1.41 6.35)	1.84** (1.25 2.72)	1.56* (1.06 2.29)

* *p* < 0.05, **p < 0.01; ***p < 0.001; UOR, unadjusted odds ratio; AOR, adjusted odds ratio; Ref, reference category; AOR, adjusted for age, sex, education, working, marital status, residence, MPCE, religion, caste, region, physical inactivity, smoking, chewing tobacco, alcohol consumption, body mass index, ADL and IADL.

Our finding further shows that individuals with family medical history had higher odds of chronic heart diseases (AOR: 2.09, CI: 1.64 2.65) and stroke (AOR: 3.07, CI: 2.33 4.03) than those without family history. Moreover, individuals with parental and sibling medical history had 2.07-and 2.09-times higher odds of having chronic heart diseases, respectively, than those without parental and sibling history. Moreover, our findings demonstrate that individuals with father, mother, brother, sister medical history had significantly 2.07, 1.79, 2.31, and 1.70 times higher odds of having chronic heart diseases, respectively compared with those without family medical history.

Similarly, individuals with parental and sibling medical history had 2.76-and 3.58-times higher odds of having stroke, respectively, than those without parental and sibling history. Moreover, our findings further demonstrate that individuals with father, mother, brother, sister medical history had significantly 2.64, 2.49, 3.58, and 2.99 times higher odds of having stroke, respectively compared with those without family medical history.

**Figure 3:**
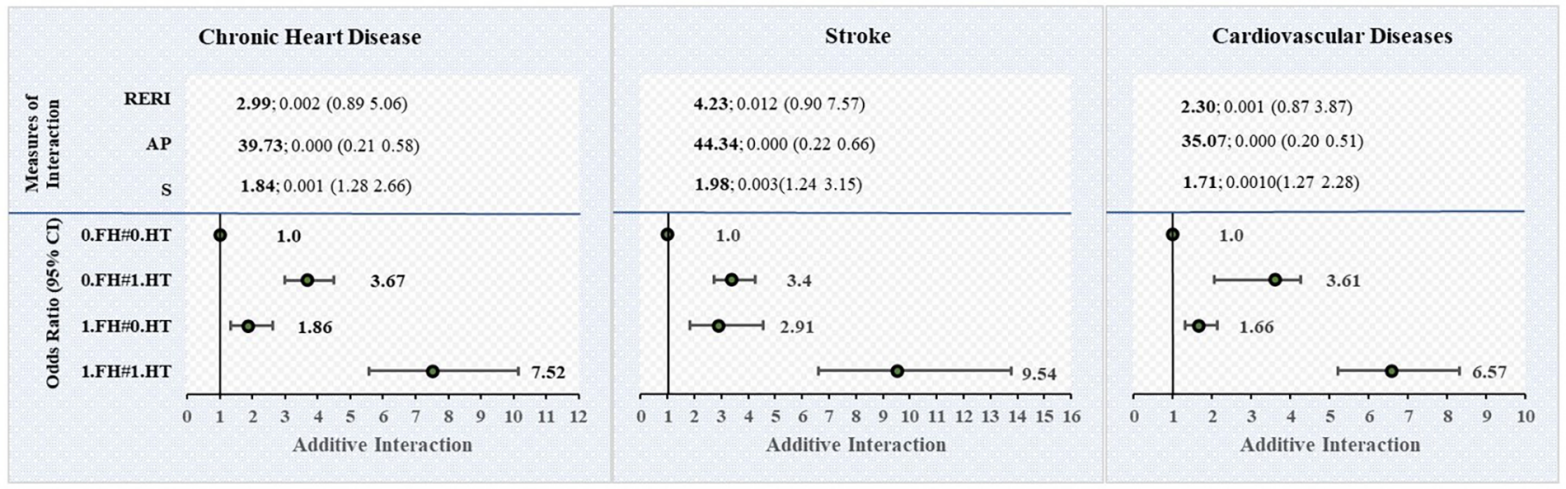
Additive interaction of family medical history of CVD with hypertension on cardiovascular diseases among individuals aged 45 and above in India. FH, family medical history (family history of chronic heart diseases, stroke, and cardiovascular diseases); HT, hypertension. Interaction exists if RERI ! = 0 or AP ! = 0 or S ! = 1. Estimates for Additive Interaction, adjusted for age, sex, education, working, marital status, residence, MPCE, religion, caste, region, physical inactivity, smoking, chewing tobacco, alcohol consumption, body mass index (BMI), ADL and IADL. RERI, Relative excess risk due to interaction; AP, Attributable proportion, AP, the attributable proportion, has been presented in the result as a percentage after multiplied by 100. S, Synergy index.

**Figure 4:**
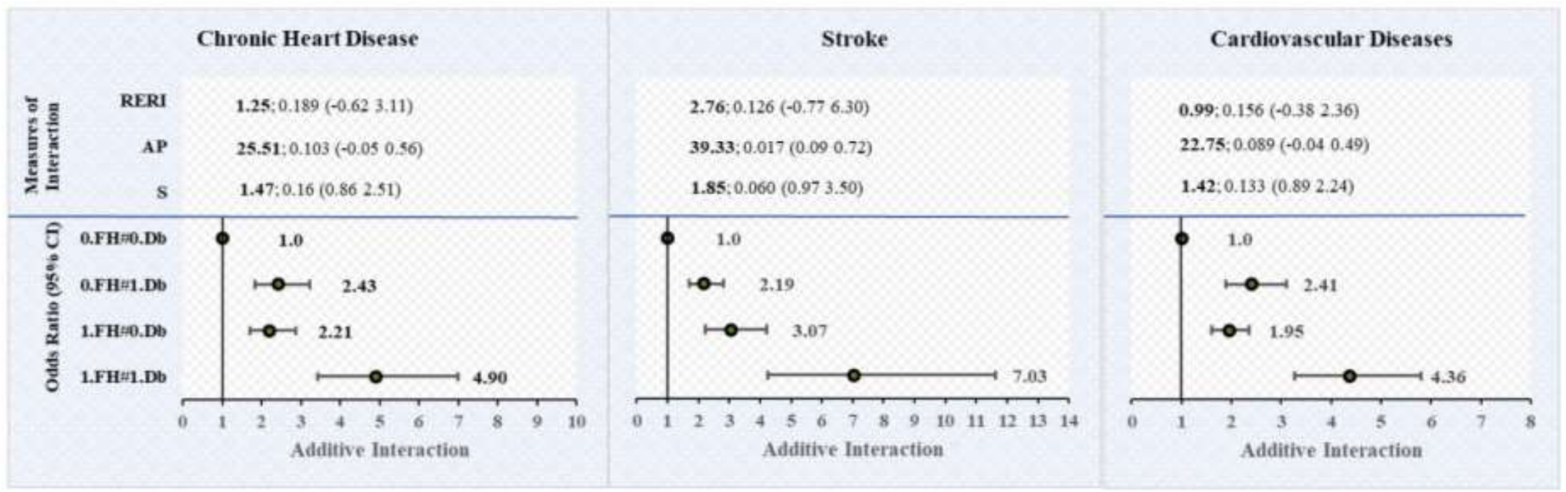
Additive interaction of family medical history of CVD with diabetes on cardiovascular diseases among individuals aged 45 and above in India. FH, family medical history (family history of chronic heart diseases, stroke, and cardiovascular diseases); Db, diabetes. Interaction exists if RERI ! = 0 or AP ! = 0 or S ! = 1. Estimates for Additive Interaction, adjusted for age, sex, education, working, marital status, residence, MPCE, religion, caste, region, physical inactivity, smoking, chewing tobacco, alcohol consumption, body mass index (BMI), ADL and IADL. RERI, Relative excess risk due to interaction. AP, Attributable proportion, AP, the attributable proportion, has been presented in the result as a percentage after multiplied by 100. S, Synergy index.

Table 5 provides the multivariable logistic regression estimates of chronic heart diseases, stroke, and CVD by the joint effect of family history of cardiovascular diseases with hypertension and diabetes. This table also provides unadjusted and adjusted models of additive interaction of family history with hypertension and diabetes on chronic heart diseases, stroke and CVD. Figure 3 shows the adjusted model of additive interaction between family medical history and hypertension on the diagnosis of cardiovascular diseases. Figure 4 shows the adjusted model of additive interaction between family medical history and diabetes on the diagnosis of cardiovascular diseases.

**Table 5:** Additive interaction of family medical history of CVD with hypertension and diabetes on cardiovascular diseases among individuals aged 45 and above in India.

	Chronic heart diseases		Stroke		Cardiovascular diseases	
	Model 1	Model 2	Model 1	Model 2	Model 1	Model 2
A | Hypertension						
Additive interaction between family history and hypertension						
0.FH#0.hypertension	Ref.	Ref.	Ref.	Ref.	Ref.	Ref.
0.FH#1.hypertension	5.42*** (4.09 7.17)	3.67*** (3.00 4.49)	4.28*** (3.48 5.28)	3.4*** (2.72 4.25)	5.08*** (4.04 6.38)	3.61*** (3.06 4.26)
1.FH#0.hypertension	2.17*** (1.56 3.02)	1.86*** (1.32 2.61)	3.16*** (2.03 4.90)	2.91*** (1.85 4.57)	1.82*** (1.43 2.33)	1.66*** (1.30 2.12)
1.FH#1.hypertension	11.19*** (8.58 14.58)	7.52*** (5.57 10.15)	12.87*** (9.16 18.09)	9.54*** (6.62 13.76)	9.10*** (7.40 11.19)	6.57*** (5.20 8.31)
Measures of interaction on an additive scale (*P*-value, 95% CI)						
RERI	**4.60**; 0.002 (1.66 7.54)	**2.99**; 0.005 (0.89 5.06)	**6.43**; 0.003 (2.23 10.62)	**4.23**; 0.012 (0.90 7.57)	**3.18**; 0.001 (1.26 5.11)	**2.30**; 0.001 (0.87 3.74)
AP^$^	**41.10**; 0.000 (0.22 0.60)	**39.73**; 0.000 (0.21 0.58)	**49.94**; 0.000 (0.31 0.69)	**44.34**; 0.000 (0.22 0.66)	**35.02**; 0.000 (0.19 0.51)	**35.07**; 0.000 (0.20 0.51)
S	**1.82**; 0.001 (1.27 2.61)	**1.84**; 0.001 (1.28 2.66)	**2.18**; 0.000 (1.44 3.31)	**1.98**; 0.003 (1.24 3.15)	**1.65**; 0.001 (1.23 2.21)	**1.71**; 0.000 (1.27 2.28)
B | Diabetes						
Additive interaction between family history and diabetes						
0.FH#0.diabetes	Ref.	Ref.	Ref.	Ref.	Ref.	Ref.
0.FH#1.diabetes	4.10*** (2.60 6.45)	2.43*** (1.84 3.23)	2.82*** (2.22 3.58)	2.19*** (1.70 2.82)	3.77*** (2.58 5.52)	2.41*** (1.88 3.10)
1.FH#0.diabetes	2.53*** (1.97 3.25)	2.21*** (1.70 2.87)	3.43*** (2.54 4.64)	3.07*** (2.23 4.23)	2.12*** (1.76 2.56)	1.95*** (1.61 2.35)
1.FH#1.diabetes	8.22*** (5.92 11.43)	4.90*** (3.42 6.99)	9.69*** (5.99 15.68)	7.03*** (4.24 11.63)	6.71*** (5.18 8.69)	4.36*** (3.27 5.80)
Measures of interaction on an additive scale (*P*-value, 95% CI)						
RERI	**2.59**; 0.111 (−0.60 5.79)	**1.25**; 0.189 (−0.62 3.11)	**4.44**; 0.063 (−0.23 9.12)	**2.76**; 0.126 (−0.77 6.30)	**1.82**; 0.103 (−0.37 4.01)	**0.99**; 0.156 (−0.38 2.36)
AP^$^	**31.60**; 0.049 (0.00 0.63)	**25.51**; 0.103 (−0.05 0.56)	**45.82**; 0.001 (0.18 0.74)	**39.33**; 0.017 (0.09 0.72)	**27.11**; 0.057 (−0.01 0.55)	**22.75**; 0.089 (−0.04 0.49)
S	**1.56**; 0.109 (0.91 2.69)	**1.47**; 0.16 (0.86 2.51)	**2.04**; 0.016 (1.14 3.67)	**1.85**; 0.060 (0.97 3.50)	**1.47**; 0.110 (0.92 2.35)	**1.42**; 0.133 (0.89 2.24)

* *p* < 0.05, **p < 0.01; ***p < 0.001; Ref, reference category; FH, family medical history (family history of chronic heart diseases and stroke, cardiovascular diseases).

Interaction exists if RERI ! = 0 or AP ! = 0 or S ! = 1.

Model 1: Unadjusted model.

Model 2: Adjusted for age, sex, education, working, marital status, residence, MPCE, religion, caste, region, physical inactivity, smoking, chewing tobacco, alcohol consumption, body mass index (BMI), ADL and IADL.

RERI, Relative excess risk due to interaction.

AP, Attributable proportion, AP^$^, the attributable proportion, has been presented in the result as a percentage after multiplied by 100, S, Synergy index.

RERI, AP, and S values have been presented in bold.

### Interaction effect of family history and hypertension on chronic heart diseases, stroke, and CVD

Our results show that the AOR of the joint effect between family history and hypertension on the diagnosis of CVD was 6.57 (95% CI: 5.20–8.31). In the adjusted model, the relative excess risk due to interaction (RERI) value for CVD was 2.30 (95% CI: 0.87–3.74), which indicates that there is a significant positive interaction between family history and hypertension on the diagnosis of cardiovascular diseases. The attributable proportion due to interaction (AP) value was 35% (0.35; 95% CI: 0.20–0.51), which suggests that a significant proportion of CVD cases in the population can be attributed to the interaction between family history and hypertension. The synergistic effect index (S) was 1.71 (95% CI: 1.27–2.28), further supporting a significant synergistic effect.

Furthermore, our findings show that the AOR of the joint effect between family history and hypertension on the diagnosis of chronic heart diseases and stroke were 7.52 (95% CI: 5.57–10.15) and 9.54 (95% CI: 6.62–13.76). In the adjusted model, the RERI values for chronic heart diseases and stroke were 2.99 (95% CI: 0.89–5.06), and 4.23 (95% CI: 0.90–7.57), respectively, which indicates that there is a significant positive interaction between family history and hypertension on the diagnosis of chronic heart diseases and stroke. The AP values were 39% (0.39; 95% CI: 0.21–0.58) and 44% (0.44; 95% CI: 0.22 0.66), which suggests that a significant proportion of chronic heart diseases and stroke cases in the population can be attributed to the interaction between family history and hypertension. Additionally, the S values were 1.84 (95% CI: 1.28–2.66) and 1.98 (95% CI: 1.24–3.15), respectively, which further supports a significant synergistic effect.

The RERI, AP and S all present consistent results as they all show significant positive interaction effect on an additive scale, which demonstrates that the combined effect is more than the sum of the individual effects of family history and hypertension on the risk of developing chronic heart diseases, stroke and CVD among older adults aged 45 years and above in India. The result also shows that the interaction effect is higher for stroke than chronic heart diseases.

### Subgroup analysis

We performed subgroup analysis based on age, gender, residence and education analysis to measure the interaction effect of family medical history and hypertension on cardiovascular diseases (Supplementary Table S1). Interestingly, our results demonstrated significant positive interaction effects between family medical history and hypertension on cardiovascular diseases on an additive scale among male respondents [chronic heart diseases[RERI: 2.27, AP: 35.76%, S: 1.74], stroke [RERI: 5.19, AP: 50.52%, S: 2.27], and CVD [RERI: 2.51, AP: 38.98%, S: 1.86]], female respondents [chronic heart diseases [RERI: 4.21, AP: 44.96%, S: 2.01], and CVD [RERI: 2.31, AP: 33.13%, S: 1.63]], those aged 55–64 years [CVD (RERI: 2.48, AP: 34.34%, S: 1.66)], urban residents [chronic heart diseases [RERI: 3.66, AP: 50.76%, S: 2.43], stroke [RERI: 6.66, AP: 55.35%, S: 2.52], and CVD [RERI: 3.14, AP: 47.03%, S: 2.24]], no education [stroke (RERI: 8.70, AP: 63.33%, S: 3.16)], secondary education [chronic heart disease (RERI: 4.25, AP: 47.44%, S: 2.15)] and higher education [CVD (RERI: 6.44, AP: 61.75%, S: 3.15)].

### Interaction effect of family history and diabetes on chronic heart diseases, stroke and CVD

The results suggest that the AOR of the joint effect between family history and diabetes on the diagnosis of chronic heart diseases, stroke and CVD were 4.9 (95% CI: 3.42 6.99), 7.03 (95% CI: 4.24 11.63) and 4.36 (95% CI: 3.27 5.80). However, RERI, AP, and S showed inconsistent results with 95% CI significance in the unadjusted and adjusted model on chronic heart diseases, stroke and CVD. In the adjusted model on chronic heart diseases, our finding shows consistent results, RERI = 1.25 (95% CI: −0.062 3.11), A*P* = 25% (0.25; 95% CI: −0.05 0.56), and S = 1.47 (95% CI: 0.86 2.5), indicating positive but insignificant interaction on an additive scale. Similarly, in the adjusted model on CVD, our finding shows insignificant results, RERI = 0.99 (95% CI: −0.062 2.36), AP = 22.75% (0.2575; 95% CI: −0.04 0.49), and S = 1.32 (95% CI: 0.89 2.24), indicating insignificant interaction on an additive scale.

Moreover, our finding shows inconsistent results on stroke, RERI = 2.76 (95% CI: −0.77 6.30), AP = 39% (0.39; 95% CI: 0.09 0.72), and S = 1.85 (95% CI: 0.97 3.67), with one measure AP, suggesting positive and significant interaction effect on stroke, which suggests that a significant proportion of stroke cases in the population can be attributed to the interaction between family history of stroke and diabetes.

### Subgroup analysis

We performed subgroup analysis based on age, gender, residence and education analysis to measure the interaction effect of family medical history and diabetes on cardiovascular diseases (Supplementary Table S2). Interestingly, our results revealed significant positive interaction effects between family medical history and diabetes on cardiovascular diseases on an additive scale among male respondents (CVD) and those aged 55–64 years (CVD).

### Male respondents and those aged 55–64 years

The results showed that in the adjusted model, among male respondents and individuals aged 55–64 years, the RERI values for CVD were 1.64 (95% CI: 0.02–3.26) and 3.16 (95% CI: 0.67–5.65), respectively, which indicates that there is a significant positive interaction between family history and diabetes on the diagnosis of cardiovascular diseases. The AP values were 38.43% (0.38; 95% CI: 0.13–0.64), and 53.38% (0.53; 95% CI: 0.32–0.75), respectively, which suggests that a significant proportion of CVD cases in the population can be attributed to the interaction between family history and diabetes. The S values were 2.01 (95% CI: 1.14–3.55), and 2.80% (2.80; 95% CI: 1.50–5.20), respectively, further supporting a significant synergistic effect. This means that the combined effect of family medical history and diabetes on the risk of developing CVD is greater than the sum of their individual effects among male respondents and those aged 55–64 years.

## Discussion

The current study presents the interaction effect of family medical history of cardiovascular diseases with hypertension and diabetes on the diagnosis of cardiovascular diseases. The study revealed a significant positive interaction on an additive scale between family medical history of cardiovascular diseases and hypertension on cardiovascular diseases as observed through all three measures (RERI, AP and S), even after adjustment with lifestyle and socio-demographic factors.

Our findings show that the prevalence of chronic heart diseases (8.0%) and stroke (4.9%) were more than two times higher among those who had family medical history compared to those without family medical history of chronic heart disease (3.5%) and stroke (1.5%) among older adults aged 45 years and above in India. A recent study showed that the prevalence of chronic heart disease was higher among individuals with family medical history (14). Blood relatives commonly share genetic predispositions to conditions like high blood pressure, heart disease, or stroke. Genes, hereditary units passed down from parents to children, can contribute to this predisposition (37). Our finding shows that individuals with family medical history had higher odds of chronic heart diseases and stroke than those without family medical history. A recent study demonstrated that the odds of heart disease were higher among individuals with family medical history (14). Consistently, one previous study demonstrated that individuals with a family history had higher odds of cardiovascular diseases (38).

The current study further demonstrates that individuals with parental and sibling medical history had two times higher odds of having chronic heart diseases and strokes, respectively than those without parental and sibling history. A previous study demonstrated that participants with at least one parent with premature cardiovascular disease had a higher risk of CVD events than those without a parental history of cardiovascular disease (39). Moreover, our findings present that individuals with paternal and maternal medical history had nearly 2.6- and 2.5-times higher odds of having stroke, respectively, compared with those without family medical history. Concordantly, one prior study presented that positive paternal family history was associated with a twofold increase in stroke risk, while maternal family history increased the risk by approximately 40% (2).

Moreover, significant modifiable risk factors for CVD are hypertension, smoking, diabetes mellitus, and lipid abnormalities (16). Our results show that the prevalence of CVD was higher among individuals with hypertension (12.4%) and diabetes (13.6%) compared to those without hypertension (2.6%) and without diabetes (4.1%). Previous studies reported that diabetes is a well-established risk factor for cardiovascular diseases, including coronary heart disease, heart failure, peripheral artery disease and stroke (40). On the other hand, hypertension is associated with the strongest evidence of causation and is highly prevalent (16).

Interaction refers to the situation in which the effect of one exposure on the outcome depends on the level of another exposure (33, 34, 41). Our findings indicate that the prevalence of CVD was higher among hypertensive individuals with family medical history of CVD (18.6%) than individuals without the coexistence of family history of CVD and hypertension (4.7%) and hypertensive individuals without family history of CVD (11.3%). One prior study demonstrated that hypertensive individuals with a positive family history of CVD had a nearly twofold higher prevalence of vascular disease compared to hypertensive individuals without a family history of CVD (20). Further, our results show that there is a significant positive interaction on the additive scale between family history of cardiovascular diseases and hypertension on the diagnosis of cardiovascular diseases, which shows that the combined effect is more than the sum of the individual effects of family history and hypertension on the risk of developing cardiovascular diseases among older adults aged 45 years and above in India. The result also shows that the interaction effect is higher for stroke than chronic heart disease. A previous study revealed that among individuals with hypertension, a family history of CVD was significantly associated with a higher prevalence of non-stroke CVD and stroke (20).

Interestingly, our finding shows inconsistent results on stroke, with one measure AP (39.0%), suggesting positive and significant interaction effect of family medical history and diabetes on stroke, which suggests that a significant proportion of stroke cases in the population can be attributed to the interaction between family history of stroke and diabetes. Moreover, subgroup analysis demonstrates the significant positive interaction effects between family medical history and diabetes on CVD on an additive scale among male respondents and those aged 55–64 years. A recent study revealed that genome-wide genetic risk scores showed a stronger association with coronary artery disease (CAD) in men compared to women for both existing and newly developed cases of CAD (42). A prior study showed that in both men and women, the presence of diabetes and a family history of early coronary heart disease significantly increased the risk of developing coronary heart disease so that for individuals with diabetes and a positive family history of the disease, approximately 74% of coronary heart disease cases could be attributed to the interaction between these factors (43).

The heightened risk of heart disease linked to a family history can be attributed to common genetic, environmental, and behavioral factors (38). Epigenetic mechanisms, through various types of reactions, are recognized as key mediators of the interaction between genes and the environment, potentially explaining the association between diabetes and cardiovascular disease (44). In the case of hypertension, epigenetic changes are influenced by intrauterine environmental factors that may impair nephron development, as well as by factors affecting autonomic responsiveness, vascular remodeling, salt sensitivity, and the renin-angiotensin system (44).

### Implication for policy, practice and future research

It has been established that a positive family history of CVD is an independent predictor of both myocardial infarction (45, 46) and stroke (47–49). Including both hypertension and family history in prognostic models for stroke leads to higher predictive value compared to models that consider only hypertension or family history alone (48). A familial medical history of premature hypertension and cardiovascular diseases is considered a crucial initial indicator of a genetic predisposition to hypertension and CVD. This circumstance may warrant clinically indicated genetic testing (15). Among individuals with higher genetic predisposition, preventive strategies may confer substantial benefits. A previous study reported that among individuals at higher genetic risk, adherence to a favorable lifestyle was found to be associated with approximately 50% lower relative risk of coronary heart disease compared to those who adhered to an unfavorable lifestyle (50). Previous studies present considerable evidence supporting the benefit of blood pressure-lowering medication in the prevention of atherosclerotic cardiovascular disease among adults with moderate to high cardiovascular risk and systolic blood pressure (SBP) ≥ 130 mm Hg or diastolic blood pressure (DBP) levels ≥80 mm Hg. These studies reveal a substantial reduction in adverse outcomes, including stroke, heart failure, coronary events, and mortality (18, 51, 52).

The study's outcomes hold significant implications for the standard clinical care of individuals diagnosed with cardiovascular diseases (CVD). Accordingly, it is advised to conduct thorough monitoring of patients exhibiting blood pressure readings nearing the upper threshold of the normal range, particularly those with a family history of CVD. As a result, concerted efforts should be undertaken to proactively prevent blood pressure elevation in this specific population.

### Limitations and strengths

The cross-sectional nature of the study precludes the establishment of causal relationship. However, it is crucial to emphasize that this is a cross-sectional study, and our findings rely on self-reported information regarding hypertension, diabetes, cardiovascular diseases and family medical history. There is a possibility of recall bias that cannot be entirely eliminated. The current study also provides the information about those who were on medication for cardiovascular diseases, which minimizes the recall bias. It is essential to recognize these limitations while interpreting the findings of this study. Despite the limitations, the current study has potential strengths. In LASI, for those who reported that they have been diagnosed with a disease by a medical professional, a set of additional questions relating to the diagnosing physician, the date of diagnosis, and if currently taking treatment were asked. This is the first population-based cross-sectional study with a large sample size that investigated the additive interaction of family medical history of cardiovascular diseases with hypertension and diabetes on cardiovascular diseases. Additional research using robust study designs, such as prospective cohort studies or randomized controlled trials, is needed to investigate further and validate the observed additive interactions.

## Conclusions

The present study revealed that in the additive model, the interaction effects of family medical history and hypertension were significantly positive on cardiovascular diseases. A family medical history of CVD can be used as a valuable tool to identify hypertensive individuals at a higher risk. Understanding the interaction between family history of cardiovascular diseases and hypertension is crucial for effective risk assessment, prevention, and team-based management strategies. Further, regular cardiovascular screenings, monitoring blood pressure, and addressing modifiable risk factors are essential in managing and reducing the risk of cardiovascular disease among individuals with family medical history and diagnosed with hypertension. Consequently, a positive family history can be employed to engage immediate family members in health education initiatives and implement early interventions for improved hypertension and cardiovascular management.

## Data Availability

Publicly available datasets were analyzed in this study. The study uses secondary data which is available upon reasonable request through: https://www.iipsindia.ac.in/content/lasi-wave-i. The data are also available in the repository of the Gateway to Global Aging Data (https://g2aging.org).
