# Supplementary material for: Additive interaction of family medical history of cardiovascular diseases with hypertension and diabetes on the diagnosis of cardiovascular diseases among older adults in India
Source: Front Cardiovasc Med. 2024 Dec 5;11:1386378. doi: 10.3389/fcvm.2024.1386378 (PMC11659756; doi:10.3389/fcvm.2024.1386378)
Supplement: Supplementary file 1 [file Table1.docx]

**Subgroup Analysis -** Gender, Age Categories, Residence, Education Level

| **Supplementary Table 1 \| Subgroup analysis of Additive Interaction of family medical history of CVD with hypertension on cardiovascular diseases among individuals aged 45 years and above** | | | | | | | |
| --- | --- | --- | --- | --- | --- | --- | --- |
|  |  | **Hypertension** | | | | | |
|  |  | **CHD** |  | **Stroke** | |  | **CVD** |
| **Gender** | | | | | | | |
| ***A \| Male*** |  |  |  |  |  | |  |
| **Additive Interaction between family history and hypertension** | | | | | | | |
| 0.FH#0.hypertension | | Ref. |  | Ref. |  | | Ref. |
| 0.FH#1.hypertension | | 3.20*** (2.48 4.13) | | 3.44*** (2.58 4.59) | | | 3.28*** (2.67 4.04) |
| 1.FH#0.hypertension | | 1.87** (1.21 2.90) | | 2.64** (1.47 4.73) | | | 1.64** (1.20 2.24) |
| 1.FH#1.hypertension | | 6.34*** (4.45 9.05) | | 10.27*** (6.31 16.71) | | | 6.43*** (4.79 8.62) |
|  |  |  |  |  |  | |  |
| **Measures of interaction on an additive scale (P-value, 95% CI)** | | | | | | | |
| **RERI** |  | **2.27**; 0.039 (1.12 4.42) | | **5.19**; 0.033 (0.42 9.95) | | | **2.51**; 0.005 (0.77 4.24) |
| **AP** |  | **35.76**; 0.004 (0.11 0.60) | | **50.52**; 0.000 (0.25 0.76) | | | **38.98**; 0.000 (0.21 0.57) |
| **S** |  | **1.74**; 0.024 (1.08 2.81) | | **2.27**; 0.008 (1.24 4.18) | | | **1.86**; 0.001 (1.28 2.70) |
| ***B \| Female*** |  |  |  |  |  | |  |
| **Additive Interaction between family history and diabetes** | | | | | | | |
| 0.FH#0.hypertension | | Ref. |  | Ref. |  | | Ref. |
| 0.FH#1.hypertension | | 4.23*** (3.18 5.61) | | 3.28*** (2.31 4.66) | | | 3.96*** (3.11 5.06) |
| 1.FH#0.hypertension | | 1.93** (1.21 3.08) | | 3.16** (1.56 6.41) | | | 1.69** (1.17 2.45) |
| 1.FH#1.hypertension | | 9.36*** (6.09 14.39) | | 8.25*** (4.70 14.50) | | | 6.96*** (4.92 9.84) |
|  |  |  |  |  |  | |  |
| **Measures of interaction on an additive scale (P-value, 95% CI)** | | | | | | | |
| **RERI** |  | **4.21**; 0.023 (0.57 7.85) | | **2.81**; 0.222 (-1.70 7.32) | | | **2.31**; 0.040 (0.10 4.51) |
| **AP** |  | **44.96**; 0.000 (0.22 0.68) | | **34.04**; 0.100 (-0.07 0.75) | | | **33.13**; 0.004 (0.10 0.56) |
| **S** |  | **2.01**; 0.004 (1.24 3.26) | | **1.63**; 0.185 (0.79 3.37) | | | **1.63**; 0.019 (1.08 2.46) |
|  |  |  |  |  |  | |  |
| **Age categories** | | | | | | | |
| ***A \| 45-55 years*** | |  |  |  |  | |  |
| **Additive Interaction between family history and diabetes** | | | | | | | |
| 0.FH#0.hypertension | | Ref. |  | Ref. |  | | Ref. |
| 0.FH#1.hypertension | | 5.50*** (3.74 8.09) | | 3.92*** (2.46 6.25) | | | 4.94*** (3.50 6.96) |
| 1.FH#0.hypertension | | 2.18** (1.22 3.87) | | 3.14** (1.54 6.39) | | | 1.95** (1.28 2.96) |
| 1.FH#1.hypertension | | 9.76*** (4.64 20.52) | | 15.68*** (7.63 32.19) | | | 9.44*** (5.68 15.71) |
|  |  |  |  |  |  | |  |
| **Measures of interaction on an additive scale (P-value, 95% CI)** | | | | | | | |
| **RERI** |  | **3.09**; 0.370 (-3.67 9.84) | | **9.62**; 0.078 (-1.07 20.30) | | | **3.56**; 0.113 (-0.85 7.97) |
| **AP** |  | **31.62**; 0.201 (-0.17 0.80) | | **61.35**; 0.000 (0.33 0.90) | | | **37.71**; 0.017 (0.07 0.69) |
| **S** |  | **1.53**; 0.284 (0.70 3.42) | | **2.90**; 0.012 (1.27 6.63) | | | **1.73**; 0.058 (0.98 3.04) |
|  |  |  |  |  |  | |  |
| ***B \| 55-64 years*** | |  |  |  |  | |  |
| **Additive Interaction between family history and diabetes** | | | | | | | |
| 0.FH#0.hypertension | | Ref. |  | Ref. |  | | Ref. |
| 0.FH#1.hypertension | | 4.17*** (3.14 5.53) | | 3.33*** (2.20 5.05) | | | 3.84*** (3.00 4.93) |
| 1.FH#0.hypertension | | 1.95* (1.08 3.51) | | 3.60** (1.58 8.21) | | | 1.90** (1.26 2.88) |
| 1.FH#1.hypertension | | 8.18*** (5.35 12.51) | | 6.54*** (3.38 12.64) | | | 7.23*** (5.07 10.31) |
|  |  |  |  |  |  | |  |
| **Measures of interaction on an additive scale (P-value, 95% CI)** | | | | | | | |
| **RERI** |  | **3.06**; 0.062 (-0.15 6.27) | | **0.61**; 0.792 (-3.89 5.10) | | | **2.48**; 0.035 (0.17 4.79) |
| **AP** |  | **37.43**; 0.007 (0.10 0.65) | | **09.26**; 0.780 (-0.56 0.74) | | | **34.34**; 0.003 (0.11 0.57) |
| **S** |  | **1.74**; 0.033 (1.05 2.91) | | **1.12**; 0.790 (0.48 2.63) | | | **1.66**; 0.017 (1.09 2.53) |
|  |  |  |  |  |  | |  |
| ***C \| 65-74 years*** | |  |  |  |  | |  |
| **Additive Interaction between family history and diabetes** | | | | | | | |
| 0.FH#0.hypertension | | Ref. |  | Ref. |  | | Ref. |
| 0.FH#1.hypertension | | 3.43*** (2.50 4.71) | | 3.15*** (2.12 4.69) | | | 3.48*** (2.66 4.56) |
| 1.FH#0.hypertension | | 2.30** (1.23 4.29) | | 2.07 (0.74 5.80) | | | 1.73* (1.07 2.79) |
| 1.FH#1.hypertension | | 7.60*** (4.73 12.20) | | 8.96*** (4.64 17.30) | | | 5.98*** (4.03 8.88) |
|  |  |  |  |  |  | |  |
| **Measures of interaction on an additive scale (P-value, 95% CI)** | | | | | | | |
| **RERI** |  | **2.87**; 0.103 (-0.58 6.31) | | **4.73**; 0.111 (-1.10 10.57) | | | **1.77**; 0.126 (-0.50 4.05) |
| **AP** |  | **37.72**; 0.020 (0.06 0.70) | | **52.84**; 0.006 (0.15 0.90) | | | **29.64**; 0.047 (0.00 0.58) |
| **S** |  | **1.77**; 0.070 (0.95 3.27) | | **2.47**; 0.067 (0.94 6.49) | | | **1.55**; 0.095 (0.93 2.60) |
|  |  |  |  |  |  | |  |
| ***D \| 75+ years*** | |  |  |  |  | |  |
| **Additive Interaction between family history and diabetes** | | | | | | | |
| 0.FH#0.hypertension | | Ref. |  | Ref. |  | | Ref. |
| 0.FH#1.hypertension | | 1.80* (1.11 2.91) | | 4.04*** (2.41 6.78) | | | 2.30*** (1.54 3.42) |
| 1.FH#0.hypertension | | 1.48 (0.64 3.42) | | 2.54 (0.69 9.36) | | | 1.08 (0.53 2.18) |
| 1.FH#1.hypertension | | 5.95*** (2.89 12.25) | | 12.14*** (4.82 30.55) | | | 4.12*** (2.28 7.45) |
|  |  |  |  |  |  | |  |
| **Measures of interaction on an additive scale (P-value, 95% CI)** | | | | | | | |
| **RERI** |  | **3.67**; 0.081 (-0.45 7.80) | | **6.55**; 0.227 (-4.07 17.16) | | | **1.75**; 0.126 (-0.50 3.98) |
| **AP** |  | **61.76**; 0.000 (0.29 0.95) | | **53.95**; 0.021 (0.08 1.00) | | | **42.42**; 0.020 (0.07 0.78) |
| **S** |  | **3.88**; 0.049 (1.01 14.96) | | **2.43**; 0.126 (0.78 7.57) | | | **2.27**; 0.094 (0.87 5.94) |
|  |  |  |  |  |  | |  |
| **Residence** | | | | | | | |
| ***A \| Rural*** |  |  |  |  |  | |  |
| **Additive Interaction between family history and diabetes** | | | | | | | |
| 0.FH#0.hypertension | | Ref. |  | Ref. |  | | Ref. |
| 0.FH#1.hypertension | | 3.92*** (3.06 5.02) | | 3.18*** (2.42 4.17) | | | 3.77*** (3.10 4.58) |
| 1.FH#0.hypertension | | 2.41*** (1.60 3.63) | | 3.12*** (1.85 5.26) | | | 1.95*** (1.46 2.62) |
| 1.FH#1.hypertension | | 8.08*** (5.05 12.94) | | 8.52*** (5.33 13.63) | | | 6.55*** (4.70 9.12) |
|  |  |  |  |  |  | |  |
| **Measures of interaction on an additive scale (P-value, 95% CI)** | | | | | | | |
| **RERI** |  | **2.75**; 0.133 (-0.83 6.33) | | **3.23**; 0.106 (-0.69 7.15) | | | **1.83**; 0.073 (-0.17 3.82) |
| **AP** |  | **34.00**; 0.029 (0.03 0.65) | | **37.89**; 0.019 (0.06 0.70) | | | **27.88**; 0.017 (0.05 0.51) |
| **S** |  | **1.63**; 0.073 (0.95 2.80) | | **1.75**; 0.066 (0.96 3.19) | | | **1.49**; 0.040 (1.02 2.18) |
|  |  |  |  |  |  | |  |
| ***B \| Urban*** |  |  |  |  |  | |  |
| **Additive Interaction between family history and diabetes** | | | | | | | |
| 0.FH#0.hypertension | | Ref. |  | Ref. |  | | Ref. |
| 0.FH#1.hypertension | | 3.30*** (2.37 4.59) | | 4.06*** (2.72 6.06) | | | 3.33*** (2.47 4.48) |
| 1.FH#0.hypertension | | 1.25 (0.72 2.19) | | 2.31* (1.07 4.98) | | | 1.20 (0.78 1.85) |
| 1.FH#1.hypertension | | 7.21*** (5.01 10.38) | | 12.03*** (6.63 21.82) | | | 6.67*** (4.82 9.23) |
|  |  |  |  |  |  | |  |
| **Measures of interaction on an additive scale (P-value, 95% CI)** | | | | | | | |
| **RERI** |  | **3.66**; 0.002 (1.30 6.02) | | **6.66**; 0.040 (0.28 13.04) | | | **3.14**; 0.001 (1.22 5.06) |
| **AP** |  | **50.76**; 0.000 (0.31 0.71) | | **55.35**; 0.000 (0.29 0.81) | | | **47.03**; 0.000 (0.29 0.66) |
| **S** |  | **2.43**; 0.001 (1.43 4.14) | | **2.52**; 0.006 (1.30 4.91) | | | **2.24**; 0.001 (1.41 3.54) |
|  |  |  |  |  |  | |  |
| **Education Level** | | | | | | | |
| ***A \| No education*** | |  |  |  |  | |  |
| **Additive Interaction between family history and diabetes** | | | | | | | |
| 0.FH#0.hypertension | | Ref. |  | Ref. |  | | Ref. |
| 0.FH#1.hypertension | | 3.86*** (2.91 5.13) | | 3.02*** (2.20 4.15) | | | 3.59*** (2.85 4.52) |
| 1.FH#0.hypertension | | 2.59*** (1.64 4.10) | | 3.02** (1.47 6.21) | | | 1.97*** (1.37 2.85) |
| 1.FH#1.hypertension | | 7.55*** (3.88 14.70) | | 13.73*** (7.76 24.29) | | | 7.14*** (4.68 10.89) |
|  |  |  |  |  |  | |  |
| **Measures of interaction on an additive scale (P-value, 95% CI)** | | | | | | | |
| **RERI** |  | **2.09**; 0.394 (-2.73 6.91) | | **8.70**; 0.025 (1.12 16.27) | | | **2.58**; 0.077 (-0.28 5.44) |
| **AP** |  | **27.74**; 0.249 (-0.19 0.75) | | **63.33**; 0.000 (0.39 0.88) | | | **36.09**; 0.008 (0.09 0.63) |
| **S** |  | **1.47**; 0.319 (0.69 3.14) | | **3.16**; 0.003 (1.47 6.78) | | | **1.72**; 0.033 (1.04 2.84) |
|  |  |  |  |  |  | |  |
| ***B \| Primary Education*** | |  |  |  |  | |  |
| **Additive Interaction between family history and diabetes** | | | | | | | |
| 0.FH#0.hypertension | | Ref. |  | Ref. |  | | Ref. |
| 0.FH#1.hypertension | | 3.26*** (2.22 4.77) | | 4.08*** (2.61 6.36) | | | 3.73*** (2.70 5.14) |
| 1.FH#0.hypertension | | 1.84 (0.99 3.42) | | 3.55** (1.48 8.51) | | | 1.77* (1.11 2.83) |
| 1.FH#1.hypertension | | 7.24*** (4.38 11.97) | | 8.07*** (4.06 16.06) | | | 6.00*** (4.00 8.99) |
|  |  |  |  |  |  | |  |
| **Measures of interaction on an additive scale (P-value, 95% CI)** | | | | | | | |
| **RERI** |  | **3.14**; 0.063 (-0.17 6.46) | | **1.45**; 0.622 (-4.30 7.19) | | | **1.50**; 0.166 (-0.63 3.63) |
| **AP** |  | **43.40**; 0.004 (0.14 0.73) | | **17.90**; 0.574 (-0.45 0.80) | | | **25.03**; 0.091 (-0.040 0.54) |
| **S** |  | **2.01**; 0.036 (1.05 3.88) | | **1.26**; 0.610 (0.52 3.03) | | | **1.43**; 0.145 (0.88 2.31) |
|  |  |  |  |  |  | |  |
| ***C \| Secondary Education*** | | |  |  |  | |  |
| **Additive Interaction between family history and diabetes** | | | | | | | |
| 0.FH#0.hypertension | | Ref. |  | Ref. |  | | Ref. |
| 0.FH#1.hypertension | | 3.47*** (2.29 5.27) | | 3.05*** (1.74 5.35) | | | 3.31*** (2.28 4.81) |
| 1.FH#0.hypertension | | 1.88 (1.00 3.56) | | 2.85* (1.20 6.76) | | | 1.48 (0.93 2.37) |
| 1.FH#1.hypertension | | 8.71*** (5.23 14.51) | | 5.68*** (2.40 13.47) | | | 5.68*** (3.68 8.77) |
|  |  |  |  |  |  | |  |
| **Measures of interaction on an additive scale (P-value, 95% CI)** | | | | | | | |
| **RERI** |  | **4.35**; 0.039 (0.22 8.48) | | **0.78**; 0.743 (-3.89 5.46) | | | **1.88**; 0.11 (-0.41 4.18) |
| **AP** |  | **49.95**; 0.000 (0.22 0.78) | | **13.79**; 0.714 (-0.60 0.88) | | | **33.19**; 0.034 (0.025 0.64) |
| **S** |  | **2.30**; 0.017 (1.16 4.53) | | **1.20**; 0.733 (0.42 3.44) | | | **1.67**; 0.089 (0.93 3.03) |
|  |  |  |  |  |  | |  |
| ***D \| Higher Education*** | |  |  |  |  | |  |
| **Additive Interaction between family history and diabetes** | | | | | | | |
| 0.FH#0.hypertension | | Ref. |  | Ref. |  | | Ref. |
| 0.FH#1.hypertension | | 4.44*** (2.84 6.95) | | 4.03*** (2.07 7.83) | | | 3.73*** (2.56 5.45) |
| 1.FH#0.hypertension | | 1.27 (0.45 3.59) | | 1.11 (0.25 5.01) | | | 1.25 (0.60 2.62) |
| 1.FH#1.hypertension | | 8.96*** (5.08 15.83) | | 15.06*** (5.72 39.65) | | | 10.43*** (6.23 17.44) |
|  |  |  |  |  |  | |  |
| **Measures of interaction on an additive scale (P-value, 95% CI)** | | | | | | | |
| **RERI** |  | **4.25**; 0.080 (-0.51 9.02) | | **10.92**; 0.107 (-2.36 24.20) | | | **6.44**; 0.009 (1.62 11.25) |
| **AP** |  | **47.44**; 0.004 (0.15 0.80) | | **72.52**; 0.000 (0.47 0.98) | | | **61.75**; 0.000 (0.42 0.81) |
| **S** |  | **2.15**; 0.044 (1.02 4.51) | | **4.47**; 0.008 (1.47 13.65) | | | **3.15**; 0.000 (1.69 5.88) |

| **Supplementary Table 2 \| Subgroup analysis of Additive Interaction of family medical history of CVD with diabetes on cardiovascular diseases among individuals aged 45 years and above** | | | | | | |
| --- | --- | --- | --- | --- | --- | --- |
|  |  | **Diabetes** | | | | |
|  |  | **CHD** |  | **Stroke** |  | **CVD** |
| **Gender** | | | | | | |
| ***A \| Male*** |  |  |  |  |  |  |
| **Additive Interaction between family history and hypertension** | | | | | | |
| 0.FH#0.diabetes | | Ref. |  | Ref. |  | Ref. |
| 0.FH#1.diabetes | | 1.66*** (1.29 2.14) | | 2.37*** (1.72 3.25) | | 1.83*** (1.48 2.27) |
| 1.FH#0.diabetes | | 1.85*** (1.35 2.53) | | 3.11*** (2.02 4.79) | | 1.79*** (1.41 2.28) |
| 1.FH#1.diabetes | | 4.44*** (2.73 7.24) | | 7.59*** (3.79 15.21) | | 4.26*** (2.90 6.26) |
|  |  |  |  |  |  |  |
| **Measures of interaction on an additive scale (P-value, 95% CI)** | | | | | | |
| **RERI** |  | **1.93**; 0.078 (-0.22 4.09) | | **3.12**; 0.249 (-2.19 8.42) | | **1.64**; 0.048 (0.02 3.26) |
| **AP** |  | **43.52**; 0.004 (0.14 0.73) | | **41.02**; 0.066 (-0.03 0.85) | | **38.43**; 0.003 (0.13 0.64) |
| **S** |  | **2.28**; 0.027 (1.10 4.73) | | **1.90**; 0.151 (0.79 4.54) | | **2.01**; 0.016 (1.14 3.55) |
| ***B \| Female*** |  |  |  |  |  |  |
| **Additive Interaction between family history and diabetes** | | | | | | |
| 0.FH#0.diabetes | | Ref. |  | Ref. |  | Ref. |
| 0.FH#1.diabetes | | 3.39*** (2.30 5.01) | | 1.95** (1.29 2.94) | | 3.12*** (2.17 4.49) |
| 1.FH#0.diabetes | | 2.79*** (1.89 4.10) | | 2.77*** (1.72 4.47) | | 2.16*** (1.62 2.87) |
| 1.FH#1.diabetes | | 5.89*** (3.57 9.71) | | 7.30*** (3.55 15.01) | | 4.70*** (3.08 7.17) |
|  |  |  |  |  |  |  |
| **Measures of interaction on an additive scale (P-value, 95% CI)** | | | | | | |
| **RERI** |  | **0.71**; 0.664 (-2.50 3.92) | | **3.58**; 0.172 (-1.55 8.72) | | **0.42**; 0.718 (-1.84 2.67) |
| **AP** |  | **12.09**; 0.634 (-0.38 0.62) | | **49.03**; 0.013 (0.10 0.88) | | **08.86**; 0.700 (-0.36 0.54) |
| **S** |  | **1.17**; 0.654 (0.59 2.33) | | **2.32**; 0.073 (0.93 5.79) | | **1.27**; 0.712 (0.60 2.13) |
|  |  |  |  |  |  |  |
| **Age categories** | | | | | | |
| ***A \| 45-54 years*** | |  |  |  |  |  |
| **Additive Interaction between family history and diabetes** | | | | | | |
| 0.FH#0.diabetes | | Ref. |  | Ref. |  | Ref. |
| 0.FH#1.diabetes | | 4.21*** (2.58 6.86) | | 2.18** (1.28 3.70) | | 3.72*** (2.30 6.01) |
| 1.FH#0.diabetes | | 2.71** (1.47 5.00) | | 3.57*** (1.93 6.60) | | 2.55*** (1.70 3.82) |
| 1.FH#1.diabetes | | 3.44** (1.54 7.68) | | 11.82*** (4.07 34.31) | | 3.66*** (2.03 6.59) |
|  |  |  |  |  |  |  |
| **Measures of interaction on an additive scale (P-value, 95% CI)** | | | | | | |
| **RERI** |  | **(-2.48)**; 0.168 (-6.02 1.05) | | **7.07**; 0.264 (-5.34 19.49) | | (**-1.61**); 0.267 (-4.44 1.23) |
| **AP** |  | **(-72.23)**; 0.321 (-2.15 0.70) | | **59.84**; 0.009 (0.15 1.04) | | **(-43.95)**; 0.363 **(**-1.39 0.51**)** |
| **S** |  | **0.50**; 0.223 (0.16 1.54) | | **2.89**; 0.100 (0.82 10.22) | | **0.62**; 0.290 (0.26 1.50) |
|  |  |  |  |  |  |  |
| ***B \| 55-64 years*** | |  |  |  |  |  |
| **Additive Interaction between family history and diabetes** | | | | | | |
| 0.FH#0.diabetes | | Ref. |  | Ref. |  | Ref. |
| 0.FH#1.diabetes | | 1.95*** (1.46 2.60) | | 1.71* (1.06 2.77) | | 1.91*** (1.46 2.50) |
| 1.FH#0.diabetes | | 1.97*** (1.34 2.91) | | 2.43** (1.30 4.56) | | 1.85*** (1.38 2.50) |
| 1.FH#1.diabetes | | 5.86*** (3.28 10.48) | | 7.60*** (3.59 16.06) | | 5.92*** (3.86 9.09) |
|  |  |  |  |  |  |  |
| **Measures of interaction on an additive scale (P-value, 95% CI)** | | | | | | |
| **RERI** |  | **2.94**; 0.088 (-0.43 6.31) | | **4.45**; 0.117 (-1.12 10.03) | | **3.16**; 0.013 (0.67 5.65) |
| **AP** |  | **50.18**; 0.002 (0.19 0.81) | | **58.64**; 0.001 (0.24 0.95) | | **53.38**; 0.000 (0.32 0.75) |
| **S** |  | **2.53**; 0.023 (1.14 5.64) | | **3.08**; 0.042 (1.04 9.08) | | **2.80**; 0.001 (1.50 5.20) |
|  |  |  |  |  |  |  |
| ***C \| 65-74 years*** | |  |  |  |  |  |
| **Additive Interaction between family history and diabetes** | | | | | | |
| 0.FH#0.diabetes | | Ref. |  | Ref. |  | Ref. |
| 0.FH#1.diabetes | | 2.20*** (1.55 3.12) | | 2.20*** (1.44 3.38) | | 2.20*** (1.60 3.03) |
| 1.FH#0.diabetes | | 2.54*** (1.67 3.87) | | 2.51** (1.35 4.70) | | 1.81*** (1.30 2.53) |
| 1.FH#1.diabetes | | 4.71*** (2.45 9.06) | | 6.79*** (2.80 16.47) | | 4.18*** (2.45 7.15) |
|  |  |  |  |  |  |  |
| **Measures of interaction on an additive scale (P-value, 95% CI)** | | | | | | |
| **RERI** |  | **0.97**; 0.554 (-2.25 4.20) | | **3.08**; 0.321 (-2.99 9.15) | | **1.17**; 0.325 (-1.16 3.51) |
| **AP** |  | **20.64**; 0.474 (-0.36 0.77) | | **45.28**; 0.093 (-0.07 0.98) | | **28.08**; 0.197 (-0.15 0.71) |
| **S** |  | **1.36**; 0.517 (0.54 3.40) | | **2.13**; 0.208 (0.66 6.93) | | **1.58**; 0.263 (0.71 3.55) |
|  |  |  |  |  |  |  |
| ***D \| 75+ years*** | |  |  |  |  |  |
| **Additive Interaction between family history and diabetes** | | | | | | |
| 0.FH#0.diabetes | | Ref. |  | Ref. |  | Ref. |
| 0.FH#1.diabetes | | 1.72 (0.85 3.46) | | 2.85*** (1.58 5.14) | | 2.07** (1.22 3.50) |
| 1.FH#0.diabetes | | 2.26** (1.24 4.11) | | 4.68*** (2.20 9.97) | | 1.90** (1.18 3.05) |
| 1.FH#1.diabetes | | 4.89** (1.81 13.20) | | 1.43 (0.21 9.88) | | 1.81 (0.79 4.14) |
|  |  |  |  |  |  |  |
| **Measures of interaction on an additive scale (P-value, 95% CI)** | | | | | | |
| **RERI** |  | **1.91**; 0.445 (-2.99 6.83) | | **(-5.10)**; 0.036 (-9.86 -0.34) | | **(-1.16)**; 0.231 (-3.07 0.74) |
| **AP** |  | **39.17**; 0.246 (-0.27 1.05) | | **(-3.569**; 0.436 (-12.55 5.41) | | **(-64.46)**; 0.384 (-2.10 0.81) |
| **S** |  | **1.97**; 0.358 (0.46 8.40) | | **0.08**; 0.433 (0.00 46.53) | | **0.41**; 0.348 (0.06 2.64) |
|  |  |  |  |  |  |  |
| **Residence** | | | | | | |
| ***A \| Rural*** |  |  |  |  |  |  |
| **Additive Interaction between family history and diabetes** | | | | | | |
| 0.FH#0.diabetes | | Ref. |  | Ref. |  | Ref. |
| 0.FH#1.diabetes | | 2.10*** (1.57 2.81) | | 2.04*** (1.47 2.84) | | 2.13*** (1.67 2.72) |
| 1.FH#0.diabetes | | 2.38*** (1.68 3.38) | | 3.03*** (2.08 4.41) | | 1.91*** (1.50 2.42) |
| 1.FH#1.diabetes | | 4.93*** (2.70 9.00) | | 6.36*** (2.93 13.81) | | 4.52*** (2.89 7.08) |
|  |  |  |  |  |  |  |
| **Measures of interaction on an additive scale (P-value, 95% CI)** | | | | | | |
| **RERI** |  | **1.45**; 0.343 (-1.55 4.46) | | **2.29**; 0.364 (-2.65 7.23) | | **1.48**; 0.152 (-0.55 3.51) |
| **AP** |  | **29.47**; 0.120 (-0.16 0.75) | | **35.97**; 0.172 (-0.16 0.88) | | **32.78**; 0.043 (0.01 0.65) |
| **S** |  | **1.59**; 0.270 (0.70 3.60) | | **1.74**; 0.262 (0.66 4.61) | | **1.73**; 0.088 (0.92 3.23) |
|  |  |  |  |  |  |  |
| ***B \| Urban*** |  |  |  |  |  |  |
| **Additive Interaction between family history and diabetes** | | | | | | |
| 0.FH#0.diabetes | | Ref. |  | Ref. |  | Ref. |
| 0.FH#1.diabetes | | 2.66*** (1.80 3.91) | | 2.51*** (1.66 3.78) | | 2.64*** (1.84 3.80) |
| 1.FH#0.diabetes | | 2.06*** (1.40 3.01) | | 3.35*** (1.85 6.07) | | 2.06*** (1.51 2.81) |
| 1.FH#1.diabetes | | 5.10*** (3.33 7.81) | | 8.35*** (4.15 16.79) | | 4.53*** (3.18 6.45) |
|  |  |  |  |  |  |  |
| **Measures of interaction on an additive scale (P-value, 95% CI)** | | | | | | |
| **RERI** |  | **1.39**; 0.225 (-0.85 3.63) | | **3.49**; 0.230 (-2.21 9.20) | | **0.82**; 0.348 (-0.90 2.54) |
| **AP** |  | **27.20**; 0.133 (-0.08 0.63) | | **41.83**; 0.060 (-0.02 0.85) | | **18.19**; 0.291 (-0.16 0.52) |
| **S** |  | **1.51**; 0.202 (0.80 2.85) | | **1.90**; 0.152 (0.79 4.60) | | **1.30**; 0.341 (0.34 2.26) |
|  |  |  |  |  |  |  |
| **Education Level** | | | | | | |
| ***A \| No education*** | |  |  |  |  |  |
| **Additive Interaction between family history and diabetes** | | | | | | |
| 0.FH#0.diabetes | | Ref. |  | Ref. |  | Ref. |
| 0.FH#1.diabetes | | 2.76*** (1.75 4.34) | | 2.11*** (1.35 3.29) | | 2.56*** (1.74 3.77) |
| 1.FH#0.diabetes | | 2.60*** (1.61 4.19) | | 4.23*** (2.62 6.85) | | 2.30*** (1.69 3.14) |
| 1.FH#1.diabetes | | 4.74*** (2.21 10.17) | | 8.95*** (3.21 24.97) | | 4.12*** (2.37 7.16) |
|  |  |  |  |  |  |  |
| **Measures of interaction on an additive scale (P-value, 95% CI)** | | | | | | |
| **RERI** |  | **38.75**; 0.852 (-3.68 4.46) | | **3.61**; 0.450 (-5.77 12.99) | | **0.26**; 0.846 (-2.33 2.85) |
| **AP** |  | **08.17**; 0.842 (-0.72 0.88) | | **40.34**; 0.228 (-0.25 1.06) | | **06.23**; 0.839 (-0.54 0.66) |
| **S** |  | **1.12**; 0.848 (0.37 3.40) | | **1.83**; 0.344 (0.52 6.42) | | **1.09**; 0.843 (0.47 2.55) |
|  |  |  |  |  |  |  |
| ***B \| Primary Education*** | |  |  |  |  |  |
| **Additive Interaction between family history and diabetes** | | | | | | |
| 0.FH#0.diabetes | | Ref. |  | Ref. |  | Ref. |
| 0.FH#1.diabetes | | 1.48* (1.04 2.11) | | 1.68* (1.05 2.69) | | 1.45* (1.06 1.99) |
| 1.FH#0.diabetes | | 1.89** (1.23 2.90) | | 2.23** (1.23 4.04) | | 1.47* (1.06 2.04) |
| 1.FH#1.diabetes | | 4.53*** (2.48 8.28) | | 6.84*** (2.88 16.26) | | 4.16*** (2.62 6.62) |
|  |  |  |  |  |  |  |
| **Measures of interaction on an additive scale (P-value, 95% CI)** | | | | | | |
| **RERI** |  | **2.15**; 0.117 (-0.54 4.85) | | **3.93**; 0.189 (-1.93 9.80) | | **2.24**; 0.019 (0.37 4.11) |
| **AP** |  | **47.58**; 0.007 (0.13 0.82) | | **57.48**; 0.005 (0.17 0.98) | | **53.80**; 0.000 (0.30 0.78) |
| **S** |  | **2.57**; 0.054 (0.98 6.70) | | **3.06**; 0.072 (0.90 10.36) | | **3.43**; 0.006 (1.43 8.21) |
|  |  |  |  |  |  |  |
| ***C \| Secondary Education*** | | |  |  |  |  |
| **Additive Interaction between family history and diabetes** | | | | | | |
| 0.FH#0.diabetes | | Ref. |  | Ref. |  | Ref. |
| 0.FH#1.diabetes | | 3.59*** (2.36 5.44) | | 2.55** (1.40 4.64) | | 3.49*** (2.32 5.25) |
| 1.FH#0.diabetes | | 3.22*** (2.08 4.97) | | 2.51* (1.22 5.14) | | 2.07*** (1.44 2.96) |
| 1.FH#1.diabetes | | 5.99*** (2.97 12.09) | | 6.10** (2.07 17.96) | | 4.70*** (2.66 8.30) |
|  |  |  |  |  |  |  |
| **Measures of interaction on an additive scale (P-value, 95% CI)** | | | | | | |
| **RERI** |  | **0.19**; 0.933 (-4.20 4.58) | | **2.05**; 0.524 (-4.25 8.34) | | **0.15**; 0.919 (-2.67 2.97) |
| **AP** |  | **03.15**; 0.931 (-0.68 0.75) | | **33.55**; 0.357 (-0.38 1.05) | | **03.13**; 0.917 (-0.56 0.62) |
| **S** |  | **1.04**; 0.932 (0.43 2.52) | | **1.67**; 0.443 (0.45 6.20) | | **1.04**; 0.918 (0.48 2.25) |
|  |  |  |  |  |  |  |
| ***D \| Higher Education*** | |  |  |  |  |  |
| **Additive Interaction between family history and diabetes** | | | | | | |
| 0.FH#0.diabetes | | Ref. |  | Ref. |  | Ref. |
| 0.FH#1.diabetes | | 1.44 (0.91 2.29) | | 2.85** (1.45 5.60) | | 1.91** (1.26 2.89) |
| 1.FH#0.diabetes | | 1.48 (0.77 2.84) | | 3.25* (1.29 8.21) | | 2.37** (1.39 4.06) |
| 1.FH#1.diabetes | | 4.38*** (2.13 9.00) | | 12.16*** (3.52 42.05) | | 4.67*** (2.39 9.14) |
|  |  |  |  |  |  |  |
| **Measures of interaction on an additive scale (P-value, 95% CI)** | | | | | | |
| **RERI** |  | **2.45**; 0.115 (-0.59 5.50) | | **7.06**; 0.326 (-7.01 21.13) | | **1.39**; 0.399 (-1.84 4.63) |
| **AP** |  | **55.99**; 0.003 (0.19 0.93) | | **58.05**; 0.042 (0.08 1.09) | | **29.79**; 0.270 (-0.23 0.83) |
| **S** |  | **3.65**; 0.077 (0.87 15.28) | | **2.72**; 0.147 (0.70 10.53) | | **1.61**; 0.353 (0.35 4.40) |
